# Pharmacophore-Conditioned Diffusion Model for Ligand-Based *De Novo* Drug Design

**DOI:** 10.1021/acs.jcim.6c01046

**Published:** 2026-10-26

**Authors:** Amira Alakhdar, Barnabas Poczos, Newell Washburn, Kevin Cusack

**Affiliations:** † Department of Chemistry, 6612 Carnegie Mellon University, Pittsburgh, Pennsylvania 15213, United States; ‡ Department of Machine Learning, Carnegie Mellon University, Pittsburgh, Pennsylvania 15213, United States; § Department of Biomedical Engineering, Carnegie Mellon University, Pittsburgh, Pennsylvania 15213, United States; ∥ AbbVie Bioresearch Center, Worcester, Massachusetts 01605, United States

## Abstract

Developing bioactive molecules remains a central, time- and cost-heavy challenge in drug discovery, particularly for novel targets lacking structural or functional data. Pharmacophore modeling presents an alternative for capturing the key features required for molecular bioactivity against a biological target. In this work, we present PharmaDiff, a pharmacophore-conditioned diffusion model for 3D molecular generation. PharmaDiff employs a transformer-based architecture to integrate an atom-based representation of the 3D pharmacophore into the generative process, enabling the precise generation of 3D molecular graphs that align with predefined pharmacophore hypotheses. Through comprehensive testing, PharmaDiff demonstrates superior performance in matching 3D pharmacophore constraints compared to ligand-based drug design methods. Additionally, it achieves higher docking scores across a range of proteins in structure-based drug design, without the need for target protein structures. By integrating pharmacophore modeling with 3D generative techniques, PharmaDiff offers a powerful and flexible framework for rational drug design.

## Graphical abstract

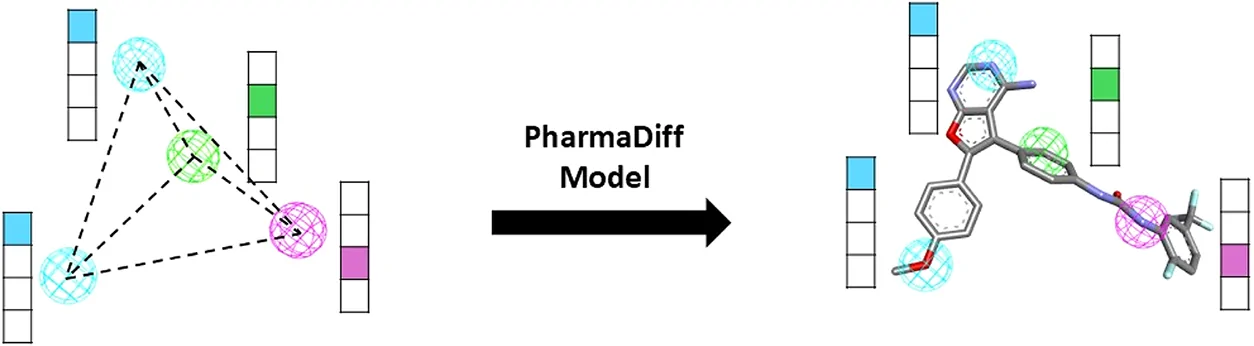

## Introduction

Computer-Aided Drug Discovery (CADD) aims to identify novel therapeutics against desired targets by investigating molecular properties using computational tools and available databases. The concept of a pharmacophore was first introduced by^1^ and is defined by IUPAC^2^ as ”an ensemble of steric and electronic features necessary to ensure optimal supramolecular interactions with a specific biological target and to trigger (or block) its biological response.” A 3D pharmacophore hypothesis also accounts for the arrangement of these chemical features relative to each other in 3D space.^3^ Pharmacophores can be either structure-based, by examining potential interactions between the macromolecular target and ligands, or ligand-based, by superposing an ensemble of active molecules and extracting consensus chemical features crucial for binding to a target.^4^ Typically, pharmacophores are used in virtual screening to filter large molecular databases such as PubChem,^5^ ChEMBL,^6^ and ZINC^7^ to identify molecules that can potentially bind to a particular target. In the virtual screening approach, pharmacophore-based filtration is usually combined with other structure-based methods such as docking and molecular dynamics, and ligand-based methods such as 2D and 3D QSARs.^4^

In parallel, deep generative models have become central to the rational design of molecules with desired properties by learning underlying data distributions. The most widely used architectures include recurrent neural networks (RNNs), variational autoencoders (VAEs), generative adversarial networks (GANs), convolutional neural networks (CNNs), and graph neural networks (GNNs). Hence, those generative models have been trained to generate molecules with desired physicochemical properties, such as the Wildman–Crippen partition coefficient (LogP), synthetic accessibility (SA), molecular weight (MW), and quantitative estimate of drug-likeness (QED).^8^ Yet, it is more challenging to design molecules with specific bioactivity profiles against therapeutic targets.

Recent advances in diffusion models have enabled the equivariant generation of 3D molecular structures.^9–11^ The Equivariant Diffusion Model (EDM)^9^ was the first to use diffusion models for 3D molecular generation, employing an E­(*n*) equivariant graph neural network (EGNN) for denoising and bond inference. Subsequent models have extended this approach with architectural innovations, including transformer, graph neural networks (GNNs), and convolutional neural network (CNN)-based architectures,^12^ most notably, MiDi,^11^ which incorporates a relaxed EGNN (rEGNN) within its E(3) graph transformer layers.

Structure-based generative models such as Pocket2Mol,^13^ GraphBP,^14^ and DiffSBDD^15^ aim to generate bioactive molecules by conditioning the generation of 3D structures of the protein’s active site. Some models combine that with other chemical or physical properties such as IPDiff^16^ that uses protein–ligand interaction priors, and MOOD^17^ that aims to generate molecules with several chemical properties, such as binding affinity, drug-likeness, and synthesizability. However, those models require the 3D structure of the active site to be available either indirectly for feature extraction or directly for conditioning the model on the 3D structure. Some models, such as Mol2Mol in REINVENT 4,^18^ constrain generation based on molecular similarity to a reference, allowing scaffold modifications; however, similarity alone may not ensure preservation of pharmacophoric features in their correct 3D arrangement.

Pharmacophore-informed generative models present an alternative approach where molecular generation is conditioned on a set of pharmacophoric features or a 3D pharmacophore hypothesis, where pharmacophores act as a bridge to connect bioactivity data to generated molecules, and several models have started adapting pharmacophoric features guidance in molecular generation. For example, a graph-based generative model called DEVELOP was developed to optimize both leads and hit-to-lead by designing linkers and R-groups guided by 3D pharmacophoric constraints.^19^ LigDream^20^ uses a variational autoencoder (VAE) to encode the 3D representation of molecules and generate molecules conditioned on molecules’ three-dimensional (3D) shape, and their pharmacophoric features. Other methods, such as TransPharmer,^21^ generate molecules satisfying the entire set of pharmacophore features of a reference compound but do not take into consideration the arrangement of the features in the 3D Euclidean space. PGMG^3^ aimed to fill that gap by generating bioactive molecules against targets using the 3D pharmacophore hypothesis. However, PGMG generates molecules represented as SMILES and replaces the 3D Euclidean distance with the shortest path distances between atoms to match the pharmacophoric hypothesis.^3^

Recent studies have further advanced conditioned 3D molecular generation through various strategies. DeepICL^22^ incorporates explicit protein–ligand interaction patterns to guide molecular generation within target binding pockets. ShEPhERD^23^ jointly models molecular shape, electrostatic surfaces, and pharmacophoric information for applications such as bioisosteric design and scaffold hopping, whereas PhoreGen^24^ employs a pharmacophore-oriented diffusion framework based on feature-level pharmacophore representations. Together, these approaches highlight the growing interest in integrating biologically meaningful constraints into molecular generation and provide complementary perspectives on guided molecular design.

In this study, we introduce a different approach to pharmacophore-conditioned molecular generation based on atom-level pharmacophore representations. Specifically, we present PharmaDiff, a pharmacophore-conditioned diffusion model that generates complete 3D molecular structures from an atom-based representation of 3D pharmacophore hypotheses (Figure 1). By bridging pharmacophore modeling with recent advances in 3D molecular generation, PharmaDiff enables pharmacophore-guided molecular design without requiring the 3D structure of the target protein.

**Figure 1:**
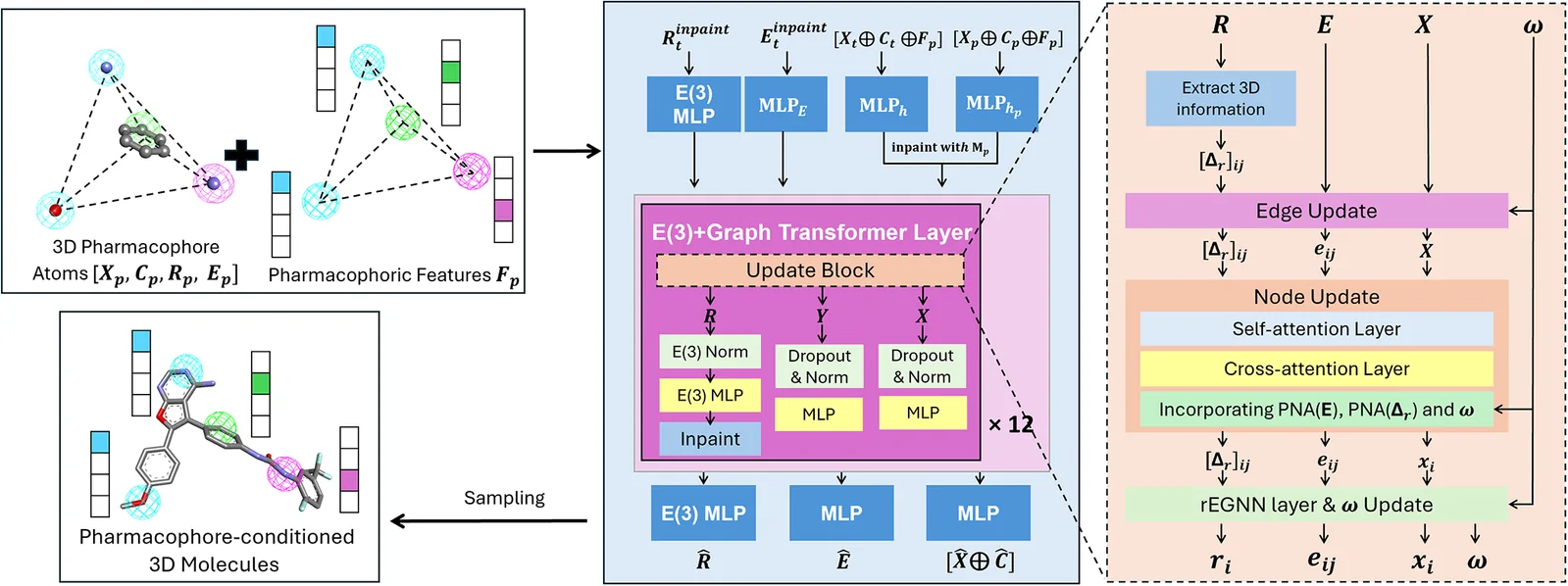
PharmaDiff’s architecture overview simultaneously predicting 2D and 3D coordinates conditioned on a pharmacophore hypothesis. The model uses 12 layers of an E(3) graph transformer architecture designed to maintain SE(3) equivariance. It incorporates inpainting and a cross-attention layer between molecular and pharmacophore node embeddings to enforce pharmacophore constraints.

## Methods

### Denoising Diffusion Models

Diffusion models are a subtype of deep generative models consisting of two Markov chains: a forward noise model and a reverse denoising model.^25,26^ The forward diffusion model is defined by transition kernels *q*(*z*_*t*_|*z*_*t*–1_) that take input data *x*_0_ ∼ *q*(*x*) and distort it by gradually injecting small amounts of noise at each time point *t* ∈ {1, 2,..., *T*} resulting in a trajectory of increasingly corrupted data points (*z*_1_, ..., *z*_*T*_) as shown in eq 1:

1 $$q\left(z_{1},...,z_{T}|x\right)=q\left(z_{1}|x\right)\prod_{t=2}^{T}q\left(z_{t}|z_{t-1}\right)$$

#### Gaussian Diffusion for Continuous Data

In the case of continuous data (such as 3D atomic coordinates), Gaussian diffusion is commonly used, where noise is added at each step via a Gaussian kernel $q\left(z_{t}|z_{t-1}\right)∼N\left(\alpha_{t}z_{t},\sigma_{t}^{2}I\right)$ . Here, σ_*t*_ determines the amount of noise injected and α_*t*_ controls the amount of signal retained. Hence, we can calculate *z*_*t*_ directly from *x* as $q\left(z_{t}|x\right)∼N\left({\mathrm{\alpha ̅}}_{t}x,{\mathrm{\sigma ̅}}_{t}^{2}I\right)$ where $\overset{®}{\alpha_{t}}≔\prod_{s=1}^{t}\alpha_{s}$ and σ̅_*t*_^2^ = σ_*t*_^2^ – α_*t*_^2^.

The reverse process is modeled by a neural network ϕ_θ_, which takes as input the noisy data *z*_*t*_, where *z*_*t*_ = α̅_*t*_ *x* + σ̅_*t*_ϵ with ϵ∼N(0,I) , and is trained to predict either the original clean data *x* or the noise ϵ that was added, rather than predicting *z*_*t*–1_ directly. This approach is commonly used in diffusion models,^25–27^ because in that case the predicted data is independent of the sampled diffusion trajectory. In the case of Gaussian diffusion, the sequence trajectories are predicted as shown in eq 2:

2 $$q\left(z_{t-1}|z_{t}\right)=N\left(z_{t-1};\mu_{\theta }\left(z_{t},t\right)x,\Sigma_{\theta }\left(z_{t},t\right)\right)$$

where θ denotes model parameters and the mean μ_θ_(*x*_*t*_, *t*) and variance Σ_θ_(*x*_*t*_, *t*) are parametrized by deep neural networks.

#### Discrete Diffusion for Categorical Data

Several studies^11,28^ suggest that discrete state-space diffusion is more suitable for categorical data (e.g., atom types or chemical bond types) where each state *z*_*t*_ is a one-hot encoded vector representing categorical distribution over the *d* possible classes (e.g., atom classes), and the forward diffusion gradually perturbs the discrete labels using a transition matrix **Q**_*t*_ ∈ *R* ^*d*×*d*^ defining the transition probabilities between discrete states. The transition kernel becomes $q\left(z_{t}|z_{t-1}\right)∼C\left(z_{t-1}Q_{t}\right)$ , and therefore, *z*_*t*_ can be calculated from *x* as *q*(*z*_*t*_ = *j*|*x*) = [*x* **Q̅**_*t*_]_*j*_, where **Q̅**_*t*_ = **Q**_**1**_ **Q**_**2**_···**Q**_*t*_. In the reverse direction, the model predicts the sequence trajectories as shown in eq 3:

3 $$q\left(z_{t-1}|z_{t}\right)\propto \sum_{x}p_{\theta }\left(x\right)\left(z_{t}Q_{t}^{\prime }⊙x{\mathrm{Q̅}}_{t-1}\right)$$

where **Q̅**_*t*_ = **Q**_**1**_ **Q**_**2**_···**Q**_*t*_, **Q**′ denotes the transpose, and ⊙ is a pointwise product.

### Diffusion for Molecules

#### Molecular Representation

A molecule is represented as a graph *G* = (**X, C, R, E**), where **X** ∈ *R* ^*n*×*d*^ is a discrete vector containing the atom type of *n* atoms belonging to *d* classes, **C** ∈ *R* ^*n*×*a*^ is also a one-hot encoded vector containing formal charges associated with *n* atoms over *a* classes, **R** ∈ *R* ^*n*×3^ represents the coordinate vectors of the *n* atoms, and **E** ∈ *R* ^*n*×*n*×*b*^ contains the adjacency matrix of the one-hot bond types encoded in the *b* classes of bond types.

#### Noise Model

We follow the same noise model as MiDi^11^ where Gaussian noise within the zero center-of-mass (CoM) is applied to the atomic coordinates satisfying ∑_*i*=1_^*n*^ ϵ_*i*_ = 0, which ensures roto-translation equivariance. Discrete diffusion is employed for all the discrete variables, including atom types, formal charges, and bond types. Hence, the noise model can be defined as shown in eq 4:

4 $$q\left(G^{t}|G^{t-1}\right)∼N^{\mathrm{CoM}}\left(\alpha^{t}R^{t-1},{\left(\sigma^{t}\right)}^{2}I\right)\times C\left(X^{t-1}Q_{x}^{t}\right)\times C\left(C^{t-1}Q_{c}^{t}\right)\times C\left(E^{t-1}Q_{e}^{t}\right)$$

We also adapt the same adaptive noise schedules as in MiDi,^11^ where the noise scheduler is manipulated, allowing for atom coordinates and bond types to be generated earlier during sampling, while atom types and formal charges are updated later in the process.

### Featurization of Pharmacophores

A pharmacophore hypothesis *G*_*p*_ consists of chemical features and their spatial distribution in 3D space. Pharmacophoric features were represented with *n* one-hot-encoded discrete vectors **F**_**p**_ ∈ *R* ^*n*×*e*^, containing the feature type associated with each atom over *e* classes of feature types. Moreover, each chemical feature is associated with one or more atoms from the molecule. For example, a hydrogen bond donor feature is associated with a single oxygen or nitrogen atom responsible for the interaction, and an aromatic feature is associated with the atoms of the aromatic ring (e.g., the six carbons of a benzene ring) and the bonds between them without any information about neighboring atoms, as shown in Figure 2A. To help the model learn the association between the chemical features and those atoms, node features (atom types **X**_**p**_ and charges **C**_**p**_), edge features **E_P_**, and positions **R**_**p**_ of atoms associated with the selected pharmacophoric features are stored as a submolecular pharmacophore graph *G*_*p*_ = (**X**_**p**_, **C**_**p**_, **F**_**p**_, **R**_**p**_, **E**_**p**_), and given as an input to the model along with the one-hot encoded pharmacophore features. Hence, the model can infer the spatial arrangement of the pharmacophoric features from the atomic positions in the pharmacophore graph.

**Figure 2:**
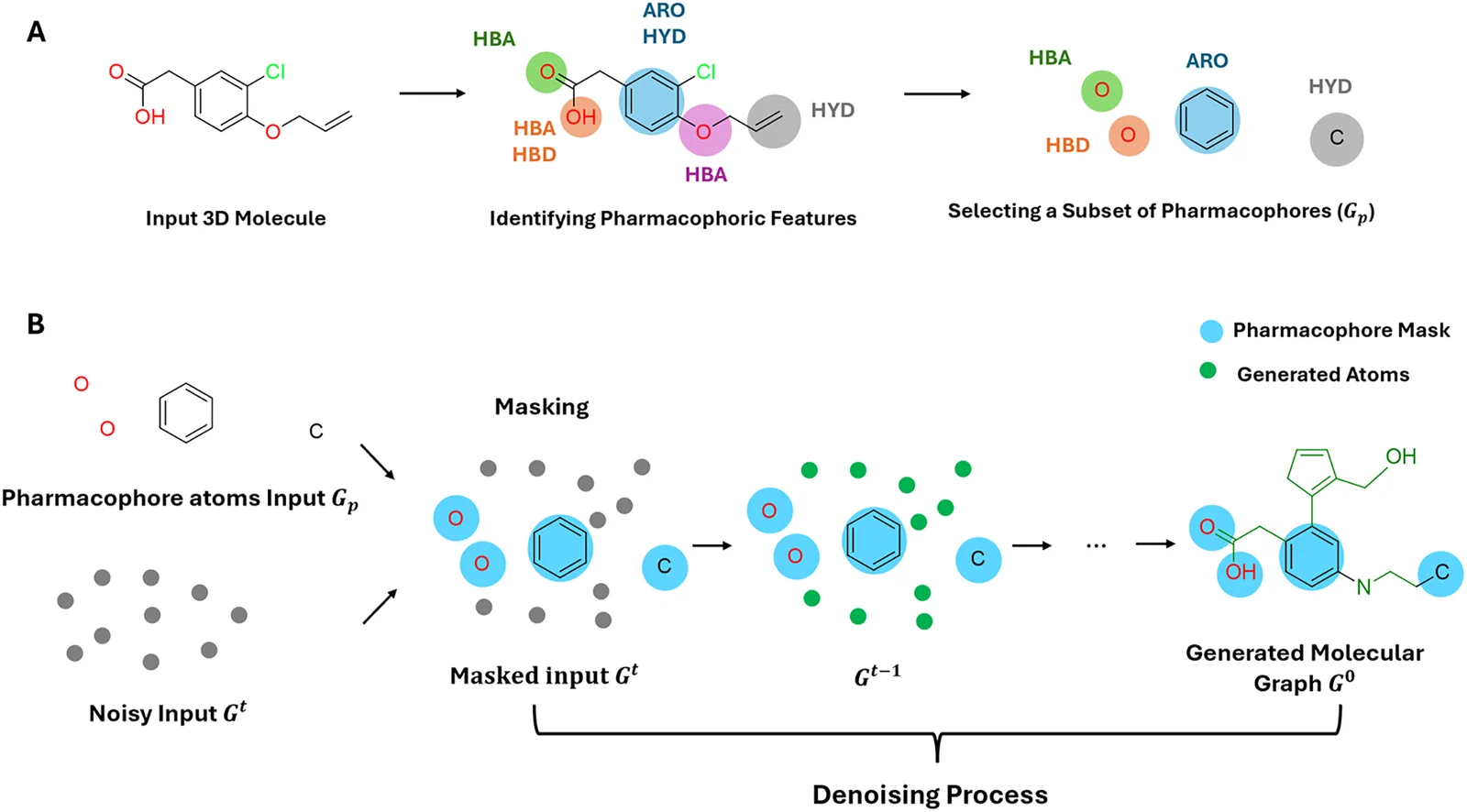
(A) Decomposition of molecular structures into 3D pharmacophore-associated atoms. (B) Conditioning the PharmaDiff model during the denoising process using the pharmacophore graph *G*_*p*_. The pharmacophoric features shown include hydrogen bond acceptor (HBA), hydrogen bond donor (HBD), hydrophobic (HYD), and aromatic (ARO) groups.

### Model Overview

PharmaDiff is a pharmacophore-conditioned, SE(3)-equivariant transformer-based diffusion model for 3D molecular generation. It builds upon the MiDi architecture,^11^ which consists of 12 SE(3)-equivariant Graph Transformer (E3-GT) layers. Each E3-GT layer includes an encoding multilayer perceptron (MLP), an update block with a self-attention module, followed by dropout, normalization layers, and decoding MLPs. PharmaDiff extends MiDi’s transformer by conditioning the generative process on a pharmacophoric graph *G*_*p*_, allowing the model to generate molecules that conform to predefined pharmacophoric patterns. This is achieved by several strategies to effectively integrate pharmacophoric information into the generation process, which will be described in this section. Also, further ablation studies are discussed in the Supporting Information.

#### Pharmacophore Feature Encoding

To incorporate pharmacophoric information, the input molecular graph is augmented with pharmacophoric features **F**_**p**_, which are concatenated with atom types **X** and formal charges **C** to form the composite node feature vector **h** = [**X**, **C**, **F**_**p**_]. This vector is then encoded using a dedicated multilayer perceptron, denoted **MLP**_**h**_, as illustrated in Figure 1. In parallel, the pharmacophore graph *G*_*p*_ is also provided as input. Its node feature vector is defined analogously as **h**_**p**_ = [**X**_**p**_, **C**_**p**_, **F**_**p**_], capturing the atom types, formal charges, and pharmacophoric descriptors associated with each pharmacophore-constrained atom. These features are encoded using a separate MLP, **MLP**_**h**_**p**__, producing pharmacophore-specific embeddings used in downstream modules such as inpainting and cross-attention.

#### Inpainting

Inpainting, also referred to as the replacement method, is employed to integrate pharmacophore-associated atoms into the noisy input. This technique is commonly used to fix atoms interacting with the protein structure, as demonstrated in structure-based models such as DiffSBDD.^15^ During the inpainting process, a predefined set of mask indices **M_p_** uniquely identifies the nodes corresponding to fixed atoms associated with the pharmacophore. These nodes’ features, embedded by **MLP**_**h_p_**_, along with their 3D positions **R**_**p**_ and edge features **E**_**p**_, are selectively replaced in the noisy input as shown in eq 5:

5 $$\left({\mathrm{MLP}}_{h}^{\mathrm{inpaint}}\left[i\right],R_{t}^{\mathrm{inpaint}}\left[i\right],E_{t}^{\mathrm{inpaint}}\left[i,j\right]\right)=\{\begin{array}{ll} \left({\mathrm{MLP}}_{h_{p}}\left[i\right],R_{p}\left[i\right],E_{p}\left[i,j\right]\right), & i\in M_{p},j\in M_{p}\\ \left({\mathrm{MLP}}_{h}\left[i\right],R_{t}\left[i\right],E_{t}\left[i,j\right]\right), & \mathrm{otherwise} \end{array}$$

This replacement mechanism ensures that the pharmacophoric substructure is explicitly injected at the beginning of each time step in the diffusion process. To further reinforce the 3D positional information on the pharmacophoric atoms throughout the network, an inpainting strategy is employed to reintroduce their positions, denoted as **R**_**p**_, at the end of each transformer layer. This repeated reinforcement enables the model to more effectively integrate spatial information with edge features, resulting in a more stable and structurally coherent molecular representation.

#### COM Adjustment

Using inpainting to introduce the 3D positional information on pharmacophoric atoms, **R**_**p**_, throughout the network can shift the center of mass (COM) and compromise the E(3)-equivariance. To prevent this, we realign the noisy atomic positions as shown in eq 6. before inpainting by translating them such that the input’s COM is centered at the origin ∑_*i*=1_^*n*^ *r*_*i*_ = 0:

6 $${\mathrm{R̃}}_{t}^{\mathrm{input}}=R_{t}^{\mathrm{input}}+\frac{1}{n}\sum_{i\in M_{p}}R_{p}-\frac{1}{n}\sum_{i\in M_{p}}R_{t,i}^{\mathrm{input}}$$

#### Cross Attention

To facilitate a more seamless and context-aware integration of pharmacophoric constraints into the molecular representation, we introduce cross-attention layers between the generated node embeddings and the pharmacophoric node embeddings. In this setup, each generated atom node (query) attends to the pharmacophoric atom nodes (keys and values), enabling the molecular representation to be dynamically influenced by the most relevant pharmacophoric features. Compared to static approaches like inpainting, this method provides a more flexible and learnable fusion of pharmacophoric information into the molecule. Formally, let $H_{\mathrm{mol}}\in R^{N\times d}$ denote the molecular node embeddings and $H_{\mathrm{pharm}}\in R^{M\times d}$ denote the pharmacophoric node embeddings. The cross-attention projections are computed as shown in eq 7:

7 $$Q=H_{\mathrm{mol}}W_{Q},,K=H_{\mathrm{pharm}}W_{K},,V=H_{\mathrm{pharm}}W_{V}$$

where $W_{Q},W_{K},W_{V}\in R^{d\times d}$ are learnable projection matrices for the **query**, **key**, and **value**, respectively. The cross-attention output is then computed as shown in eq 8:

8 $$O=W_{\mathrm{out}}\left(\mathrm{Softmax}\left(\frac{W_{A}\left({\mathrm{QK}}^{T}\right)}{\sqrt{d}}\right)V\right)$$

Here $W_{\mathrm{out}}\in R^{d\times d}$ and $W_{A}\in R^{d\times d}$ are additional learnable projections. The output is **O** is then used to inpaint **H**_mol_.

#### Loss Function

Following MiDi’s training objective, our denoising network is trained to recover the original molecular structure *G* = (**X, C, R, E**) from a noisy input *G*_*t*_. As shown in the loss function, the prediction of atomic coordinates **R̂** is treated as a regression problem, optimized using mean-squared error (MSE), while the predictions for atom types (**X̂**), formal charges (**Ĉ**), and bond types (**Ê**) are optimized as classification problems, trained with cross-entropy loss (CE).

However, unlike MiDi, our model explicitly incorporates pharmacophoric constraints to ensure that the generated molecular structure adheres to the predefined pharmacophoric features. To this end, we place greater emphasis on the accurate prediction of pharmacophoric atom types **X**_**p**_, formal charges **C**_**p**_, and positions **R**_**p**_ compared to the rest of the molecular atoms. This is achieved through an additional pharmacophore loss term, which imposes specific position, atom type, and charge constraints on the atoms associated with the pharmacophore features. The final loss function is formulated as shown in eq 9:

9 $$l\left(G,Ĝ\right)=\underset{m\mathrm{olecular}\;\mathrm{generation}\;\mathrm{loss}}{\underset{︸}{\lambda_{r}∥\mathrm{R̂}-R{∥}^{2}+\lambda_{x}\mathrm{CE}\left(X,\mathrm{X̂}\right)+\lambda_{c}\mathrm{CE}\left(C,Ĉ\right)+\lambda_{e}\mathrm{CE}\left(E,Ê\right)}}+\underset{p\mathrm{harmacophore}\;\mathrm{loss}}{\underset{︸}{\lambda_{r_{p}}∥\mathrm{R̂}-R_{p}{∥}^{2}+\lambda_{x_{p}}\mathrm{CE}\left(X_{p},\mathrm{X̂}\right)+\lambda_{c_{p}}\mathrm{CE}\left(C_{p},Ĉ\right)}}$$

Here, λ_*r*_, λ_*x*_, λ_*c*_, λ_*e*_, λ_*r*_*p*__, λ_*x*_*p*__, and λ_*c*_*p*__ are weighting factors that control the relative importance of different components of the loss. These hyperparameters were selected based on a series of short numerical experiments. Although pharmacophore-associated atoms are included in the general molecular generation loss, they typically constitute only a small fraction of the molecular graph. Consequently, their contribution to the overall optimization objective may be relatively small compared to that of unconstrained atoms. The pharmacophore-specific loss therefore increases the guidance signal associated with these atoms and encourages more accurate preservation of their positions, atom types, and charges during generation. Ablation studies evaluating the contribution of this loss component are provided in the Supporting Information.

### Dataset

The model was trained on the GEOM-DRUGs dataset,^29^ which is well-suited for drug design applications, as it contains over 450,000 drug-sized molecules with an average of 44.4 atoms (24.9 heavy atoms) and a maximum of 181 atoms (91 heavy atoms). The dataset was split into 80% for training, 10% for validation, and 10% for testing. For each molecule, the five lowest-energy conformations were selected to build the dataset. For each conformer, all chemical features were identified using RDKit, and a subset of 3–7 random features was selected to build the 3D pharmacophoric hypothesis. For the structure-based drug design experiment, the structures of the targets: BRD4 (PDB ID 3MXF), VEGFR2 (PDB ID 1YWN), CDK6 (PDB ID 2EUF), and TGFB1 (PDB ID 6B8Y) were downloaded from the Protein Data Bank.^30^

### Evaluation Metrics

#### Basic Generation Metrics

The quality of the generated molecules was assessed using four core metrics. For all metrics, only the largest connected fragment of each generated molecule was used for evaluation. **Validity** was measured by the success rate of RDKit sanitization of the largest connected fragment. **Uniqueness** represents the proportion of valid molecules that have distinct canonical SMILES. **Novelty** is defined as the fraction of unique molecules whose canonical SMILES do not appear in the training set. Finally, **Diversity** was calculated as 1 – average pairwise Tanimoto similarity,^31,32^ using Morgan fingerprints^33^ (radius 2, 2048 bits). Diversity values range from 0 to 1, with the reported value representing the average across all generation conditions.

#### Pharmacophore Match Evaluation

To assess how well the generated molecules match the pharmacophore hypotheses they were conditioned on, we used two key metrics. The **Match Score (MS)**, adapted from PGMG,^3^ quantifies the degree to which molecules align with their 3D pharmacophoric hypotheses, and it ranges from 0 to 1, where an MS of 1.0 entails a perfect match. Unlike the original PGMG implementation, which uses shortest path (graph-based) distances, we computed MS using actual Euclidean distances in 3D space between atoms. The second metric, **Perfect Match Rate (PMR)**, reflects the proportion of generated molecules that achieved an MS of 1, corresponding to a perfect match (MS = 1) to the specified 3D pharmacophore hypothesis.

#### Binding Affinity Estimation

We estimated the binding affinity of generated molecules using the **Vina Score**,^34^ a scoring function from AutoDock Vina that quantifies the predicted interaction strength between ligands and protein targets in molecular docking simulations. Average Vina scores are reported for the top 1, 100, and all of the generated molecules.

#### Drug-likeness and Physicochemical Properties

To evaluate the potential bioactivity of the generated molecules, we also report several key chemical properties of the generated molecules that are particularly relevant for drug design applications. These include molecular weight, quantitative estimate of drug-likeness (QED), synthetic accessibility (SA) score,^35^ partition coefficient (logP), topological polar surface area (TPSA), and the average number of rings.

#### 3D Plausibility PoseBusters Pass Rate (PBR)

The percentage of generated molecules that pass all PoseBusters^36^ checks. The evaluated checks assess molecular connectivity, geometric validity, steric and energetic plausibility, and chemical validity, including bond lengths, bond angles, ring planarity, steric clashes, internal energy, valence consistency, sanitization, and the absence of radical species. Higher PBR values indicate greater structural plausibility and physicochemical realism of the generated 3D conformations.

### Computational Details

Dataset processing was performed in Python (v3.9) using RDKit (2022.03.2) for molecular preprocessing, chemical structure handling, pharmacophore feature extraction, MMFF minimization, and the calculation of SA, QED, and molecular fingerprints. PyTorch (v2.0.1, CUDA 11.8), PyTorch Lightning (v2.0.4), and Torch Geometric (v2.3.1) were used for model development and training. Configuration was managed with Hydra (v1.3.2) and OmegaConf (v2.3.0). Logging and experiment tracking were handled using Weights & Biases (wandb, v0.15.4). Baseline methods were obtained from their official implementations with only minimal adaptations for input/output compatibility. PGMG^3^ was used under the CC BY-NC-SA 4.0 License from https://github.com/CSUBioGroup/PGMG. REINVENT4 ^18^ (Apache 2.0) was obtained from https://github.com/MolecularAI/REINVENT4. TransPharmer^21^ and DiffSBDD^15^ were used under the MIT License from https://github.com/iipharma/transpharmer-repo and https://github.com/arneschneuing/DiffSBDD, respectively. ShEPhERD and PhoreGen^23^ were used under the MIT Licenses from https://github.com/coleygroup/shepherd and https://github.com/ppjian19/PhoreGen, respectively. Data processing was conducted on the RM-512 partition of the Bridges-2 high-performance computing (HPC) system. Model training was performed on a single NVIDIA RTX 3090 Ti GPU (24 GB VRAM) over 390 epochs, requiring approximately 16 days. Sampling 1000 molecules required 1659.63 s (27.7 min). For the pharmacophore-conditioned benchmark, a total of 25,000 molecules were generated across 250 pharmacophore conditions. The complete benchmark, including molecule generation and evaluation, required approximately 1 day. In addition, the baseline methods were executed on HPC nodes equipped with NVIDIA V100 GPUs. Molecular structures were converted from SMILES representations to 3D conformers and subsequently minimized using the MMFF force field on HPC RM nodes. For the DDR1 case study, the computational workflow consisted of LigPrep preparation, Glide molecular docking, Prime MM/GBSA rescoring, molecular dynamics simulations, WaterMap analysis, FreeForm strain analysis, and IFD-MD refinement. These calculations were performed on compute nodes equipped with dual NVIDIA RTX A5000 GPUs (NVIDIA GA102GL) and 32 CPU cores. Glide docking, including LigPrep preparation, was primarily CPU-based and typically required approximately 4–10 h for 1000 molecules and 1–2 days for 10,000 molecules. MM/GBSA optimization of the top 100 compounds was also CPU-based and required approximately 18–24 h using a 6 Å residue refinement shell. Molecular dynamics simulations required approximately 2–6 h per protein–ligand complex using two RTX A5000 GPUs, while WaterMap analyses required less than one hour per complex using the same GPU configuration. FreeForm strain analysis was typically completed within minutes for molecules with a limited number of rotatable bonds, although highly flexible molecules could require up to 1–5 h. Finally, IFD-MD refinement required approximately 36–54 h for typical DDR1 protein–ligand complexes and up to 130 h for particularly challenging systems, utilizing both CPU and GPU resources during different stages of the workflow.

## Results

### Ligand-Based Drug Design

To evaluate the effectiveness of PharmaDiff in pharmacophore-conditioned molecular generation, we randomly selected 250 compounds, referred to as conditioning compounds, along with their corresponding 3D pharmacophore hypotheses from the reserved test set of the GEOM-Drugs dataset. Each pharmacophore hypothesis consisted of 3 to 7 features, such as hydrogen bond donors, acceptors, hydrophobic groups, or aromatic rings, randomly extracted from the conditioning compounds. These hypotheses were used to guide the de novo generation of 3D molecular structures.

We compare our method with TransPharmer’s (72bit, 108bit, 1032bit) models,^21^ REINVENT 4’s Mol2Mol high and medium similarity models,^18^ PGMG,^3^ ShEPhERD^23^ and PhoreGen.^24^ For TransPharmer and REINVENT4, 100 molecules were generated per conditioning compound, each conditioned on the full set of pharmacophoric features derived from the reference compound. PGMG, by contrast, directly employs a 3D pharmacophore hypothesis to guide the generation process. For these 2D baseline methods, 3D conformations of the generated molecules were constructed from SMILES representations using RDKit’s ETKDG algorithm, followed by geometry refinement via energy minimization with the MMFF94 force field.^37^ ShEPhERD^23^ and PhoreGen^24^ are 3D molecular generative models that directly condition on pharmacophore hypotheses and produce molecules with 3D coordinates, bypassing the need for posthoc conformer generation.

For ShEPhERD, the molecular size, *N*_*x*_1__, must be specified during conditional sampling. Because the target molecular size is unknown a priori in our ligand-based generation setting, we sampled *N*_*x*_1__ from the conditional molecular-size distribution of the MOSES-aq training set, *P*(*N*_*x*_1__|*N*_*x*_4__), rather than deterministically assigning molecular size from the number of pharmacophore features. A lower bound of *N*_*x*_1__ = 45 was applied to avoid generating molecules that were too small to accommodate the spatially distributed pharmacophore features considered in this benchmark. This follows the same general strategy used in PharmaDiff, where molecular size is sampled from the training-set size distribution when the target size is not known in advance. Under this sampling configuration, ShEPhERD generated molecules containing 17.6 heavy atoms on average, with most generated structures containing 15–22 heavy atoms and a maximum of 25 heavy atoms. For this experiment, results are reported as the mean ± standard errors of per-condition values, reflecting variability across the 250 pharmacophore conditions (Table 1 and 2).

**Table 1:** General Generation Performance of Pharmacophore-Conditioned Molecular Generative Models, Results Are Reported as Mean ± Error across all 250 Conditions

metric	validity ↑	uniqueness ↑	novelty ↑	diversity ↑
PharmaDiff (3D)	0.8823 ± 0.0037	0.9933 ± 0.0020	0.9989 ± 0.0002	**0.8686** ± **0.0028**
PGMG (2D) + MMFF	0.9439 ± 0.0039	0.9945 ± 0.0007	0.9945 ± 0.0010	0.8294 ± 0.0019
TransPharmer-72bit (2D) + MMFF	**0.9922** ± **0.0013**	0.8928 ± 0.0074	0.9556 ± 0.0034	0.7503 ± 0.0049
TransPharmer-108bit (2D) + MMFF	0.9900 ± 0.0018	0.7658 ± 0.0116	0.9628 ± 0.0032	0.6702 ± 0.0074
TransPharmer-1032bit (2D) + MMFF	0.9690 ± 0.0036	0.7175 ± 0.0130	0.9790 ± 0.0021	0.6148 ± 0.0085
REINVENT4 medium similarity (2D) + MMFF	0.9710 ± 0.0068	0.9877 ± 0.0015	0.9797 ± 0.0038	0.5282 ± 0.0036
REINVENT4 high similarity (2D) + MMFF	0.9551 ± 0.0081	0.9755 ± 0.0021	0.9960 ± 0.0016	0.4210 ± 0.0034
PhoreGen (3D)	0.8704 ± 0.0649	0.9999 ± 0.0011	1.0000 ± 0.0000	0.8404 ± 0.0167
ShEPhERD (3D)	0.6523 ± 0.0700	**1.0000** ± **0.0000**	1.0000 ± 0.0000	0.8216 ± 0.0134

**Table 2:** Pharmacophore Matching and Posebuster Performance of Pharmacophore-Conditioned Molecular Generative Models

metric	MS ↑	PMR ↑	%MS ≥ 0.8 ↑	PBR (%) ↑
PharmaDiff (3D)	**0.8964** ± **0.0050**	**0.6990** ± **0.0142**	0.8050 ± 0.0094	48.68 ± 10.89
PGMG (2D) + MMFF	0.8938 ± 0.0063	0.5099 ± 0.0225	**0.8286** ± **0.0131**	-
TransPharmer-72bit (2D) + MMFF	0.8125 ± 0.0077	0.2795 ± 0.0204	0.6383 ± 0.0195	-
TransPharmer-108bit (2D) + MMFF	0.8233 ± 0.0078	0.3171 ± 0.0218	0.6570 ± 0.0199	-
TransPharmer-1032bit (2D) + MMFF	0.8313 ± 0.0078	0.3477 ± 0.0225	0.6758 ± 0.0203	-
REINVENT4 medium similarity (2D) + MMFF	0.8424 ± 0.0080	0.3826 ± 0.0234	0.7045 ± 0.0196	-
REINVENT4 high similarity (2D) + MMFF	0.8664 ± 0.0072	0.4252 ± 0.0240	0.7530 ± 0.0189	-
PhoreGen (3D)	0.7430 ± 0.2032	0.3182 ± 0.3225	0.5563 ± 0.3533	64.03 ± 8.79
ShEPhERD (3D)	0.5160 ± 0.2417	0.1206 ± 0.2255	0.2188 ± 0.2823	95.97 ± 5.90

In terms of standard molecular generation evaluation metrics, as shown in Table 1, PharmaDiff achieves near-perfect novelty (0.9989) and uniqueness (0.9933). Its validity rate of 0.8823 is lower than several of the 2D baseline models, but comparable to PhoreGen (0.8704) and higher than ShEPhERD (0.6523), and is on par with other 3D approaches, especially models using molecular inpainting or fragment-based design.^15,38^ Notably, PharmaDiff excels in diversity (0.8686), outperforming all other models and indicating a broader exploration of the chemical space. PhoreGen also demonstrates strong general generation performance, with a validity of 0.8704, uniqueness of 0.9999, novelty of 1.0000, and diversity of 0.8404. Thus, PharmaDiff and PhoreGen exhibit broadly comparable general molecular-generation performance where PharmaDiff shows slightly higher validity and diversity, whereas PhoreGen achieves marginally higher uniqueness and novelty. ShEPhERD achieves perfect uniqueness and novelty under the evaluated sampling configuration and retains high diversity (0.8216), although its validity is lower (0.6523).

In terms of molecular-pharmacophore alignment metrics (Table 2), the results show that PharmaDiff achieves the highest mean MS (0.8964), along with the highest fraction of molecules exactly matching the conditioning pharmacophores with an average **PMR** of 0.6990, representing the highest mean MS and PMR among all evaluated methods. Compared with the 2D baseline methods, PharmaDiff demonstrates stronger pharmacophore recovery overall. Similarity-driven approaches such as REINVENT4 and the general pharmacophore-matching approach used in TransPharmer achieve relatively high mean MS values, but show lower exact and near-exact pharmacophore recovery as measured by PMR and the fraction of molecules with MS ≥ 0.8. PGMG performs more competitively because it is explicitly conditioned on a specific 3D pharmacophore. However, PharmaDiff achieves a substantially higher PMR than PGMG, although PGMG achieves a slightly higher fraction of molecules with MS ≥ 0.8. PharmaDiff’s strong performance in terms of exact pharmacophore matching is likely attributed to its use of true 3D Euclidean distances for pharmacophore matching, in contrast to the graph-based shortest-path approximations employed by PGMG.

When compared with direct 3D pharmacophore-conditioned methods, PharmaDiff also demonstrates stronger pharmacophore-matching performance. PharmaDiff achieves a mean MS of 0.8964, compared with 0.7430 for PhoreGen and 0.5160 for ShEPhERD. A similar trend is observed for exact pharmacophore matching, with PMR values of 0.6990, 0.3182, and 0.1206 for PharmaDiff, PhoreGen, and ShEPhERD, respectively. Likewise, the fraction of generated molecules with MS ≥ 0.8 is 0.8050 for PharmaDiff, compared with 0.5563 for PhoreGen and 0.2188 for ShEPhERD. These results indicate that PharmaDiff more consistently satisfies the prescribed 3D pharmacophore constraints while maintaining high molecular diversity and validity.

In contrast to the pharmacophore-matching results, ShEPhERD and PhoreGen achieve higher PoseBusters pass rates (PBR) among valid generated molecules than PharmaDiff, with values of 95.97 ± 5.90% and 64.03 ± 8.79%, respectively, compared with 48.68 ± 10.89% for PharmaDiff. This suggests that the evaluated 3D models exhibit different trade-offs between pharmacophore fidelity and structural plausibility. PharmaDiff applies a stricter approach by preserving pharmacophoric atoms through atom inpainting, which improves the recovery of the conditioning pharmacophore but may limit the model’s ability to satisfy all geometric and energetic criteria assessed by PoseBusters. Nevertheless, preserving these atoms does not guarantee exact feature recovery, since PharmaDiff conditions on the atoms associated with a pharmacophore feature rather than the complete functional group responsible for that feature. Neighboring atoms and bonds introduced during generation can therefore alter the resulting pharmacophore assignment. For example, a hydrophobic atom may end up in a polar context, or a donor oxygen may be incorporated into a carbonyl rather than a hydroxyl group, thereby losing its donor character. Disconnected fragments can also occur when pharmacophore features are spatially distant and only a small number of atoms are generated to link them, further reducing perfect match rates. Overall, these results highlight the challenge of simultaneously optimizing pharmacophore matching and 3D structural plausibility in pharmacophore-conditioned molecular generation.

### Physicochemical Property Distributions of Molecules Generated by PharmaDiff

To assess the relevance of the generated molecules as potential drugs, we compared their physicochemical property distributions to those of the training data. As illustrated in Figure 3, the generated molecules closely resemble the GEOM-Drugs dataset in terms of key properties, including molecular weight (MW), Log *P*, QED, TPSA, and ring count. While PharmaDiff exhibits slightly higher synthetic accessibility (SA) scores, it remains well within the acceptable range for drug-like compounds. These results suggest that PharmaDiff effectively captures and reproduces the molecular property distribution of its training data.

**Figure 3:**
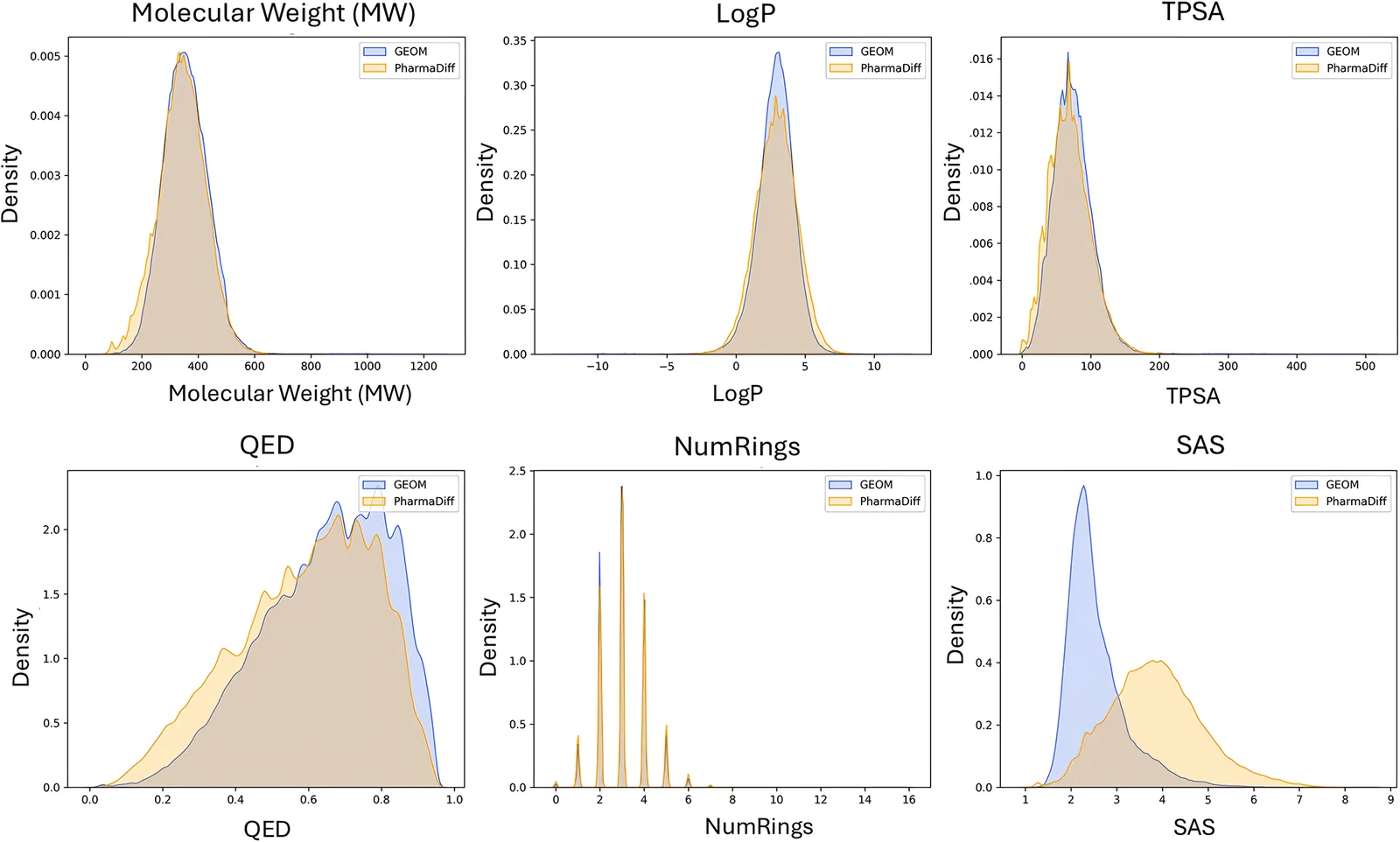
Distribution of physicochemical properties for the GEOM-Drugs training set and molecules generated by PharmaDiff. The plot compares 100,000 molecules generated from random pharmacophore hypotheses with 100,000 conformers randomly selected from the GEOM-Drugs training set.

### Structure-Based Drug Design

For the structure-based setting, we evaluated the ability of PharmaDiff to generate ligands compatible with the active sites of four proteins: BRD4, VEGFR2, CDK6, and TGFB1 with pharmacophore hypotheses reported in^3^ and collected from the literature.^39–43^ The 3D coordinates and types of pharmacophore-associated atoms were provided as fixed input. PharmaDiff was compared against DiffSBDD-cond,^15^ which uses protein structure and employs inpainting to inject information about fixed parts of the ligand into the sampling process. We provided the pharmacophore-associated atom positions and types as input for the fixed atoms. For each protein target, 1000 molecules were sampled from each model. For this experiment, results are reported as the mean ± standard errors of per-molecule values, reflecting variability across the generated molecules (Table 3).

**Table 3:** Comparison of Docking Scores, Drug-like Properties and PoseBusters Pass Rate (PBR) between PharmaDiff and DiffSBDD across Four Protein Targets, Results Are Reported as Mean ± Standard Error (SE)

		vina score (Min) ↓	vina score (Min) ↓	vina score (Min) ↓	SA score ↓	diversity ↑	MS ↑	PBR (%) ↑
protein	method	all (kcal/mol)	top 1 (kcal/mol)	top 100 (kcal/mol)	top 100	top 100	top 100	all
1ywn	PharmaDiff	**–5.7730** ± **0.0513**	**–10.6380**	**–8.3185** ± **0.0489**	**3.3236** ± **0.0560**	0.8098 ± 0.0008	**0.8840** ± **0.0150**	**26.24**
	DiffSBDD	–4.6341 ± 0.0224	–8.9850	–6.4088 ± 0.1175	3.6607 ± 0.0961	**0.8373** ± **0.0013**	0.5827 ± 0.0306	1.91
2euf	PharmaDiff	**–7.4273** ± **0.0405**	**–9.2768**	**–9.2767** ± **0.0388**	**3.2241** ± **0.0657**	0.8309 ± 0.0008	**0.9700** ± **0.0126**	**55.75**
	DiffSBDD	–7.2906 ± 0.0379	–9.0236	–9.0236 ± 0.0435	3.8148 ± 0.0781	**0.8737** ± **0.0005**	0.7833 ± 0.0290	53.01
3mxf	PharmaDiff	**–6.8463** ± **0.0355**	–10.7130	–8.5968 ± 0.0485	**3.5691** ± **0.0642**	0.8537 ± 0.0006	**0.8183** ± **0.0203**	**25.20**
	DiffSBDD	–5.7141 ± 0.0535	**–10.8790**	**–8.6539** ± **0.0640**	4.2065 ± 0.0673	**0.8830** ± **0.0005**	0.5767 ± 0.0236	24.02
6b8y	PharmaDiff	**–7.8427** ± **0.0440**	–11.4270	**–9.7503** ± **0.0485**	**3.4352** ± **0.0500**	0.8307 ± 0.0008	**0.8800** ± **0.0126**	**59.07**
	DiffSBDD	–6.2078 ± 0.0601	**–12.1900**	–9.4052 ± 0.0706	3.7569 ± 0.0800	**0.8818** ± **0.0006**	0.7217 ± 0.0250	39.83

Table 3 compares PharmaDiff and DiffSBDD across four protein targets: 1ywn (6 features), 2euf (3 features), 3mxf (4 features), and 6b8y (4 features), using docking scores, synthetic accessibility, diversity, and pharmacophore matching metrics. PharmaDiff consistently achieves better average Vina scores across all targets, and stronger top-100 docking performance in three out of the four targets, particularly excelling on 1ywn and 6b8y, which have more complex pharmacophore constraints. While Top-1 scores are more competitive, with DiffSBDD slightly outperforming on 3mxf and 6b8y, PharmaDiff still matches or exceeds performance on the remaining targets. PharmaDiff also demonstrates better synthetic feasibility, as shown by consistently lower SA scores across all targets, indicating its ability to generate realistic and accessible molecules even under stricter pharmacophore demands, such as those in 1ywn. In terms of chemical diversity, DiffSBDD holds a slight advantage across the board; however, the difference is minor, and PharmaDiff maintains high diversity while also enforcing complex constraints. Most notably, PharmaDiff significantly outperforms DiffSBDD in pharmacophore matching score (MS) across all targets, especially in 1ywn and 2euf, suggesting a higher fidelity in satisfying both simple (3 features) and more intricate (6 features) 3D pharmacophore patterns during molecule generation.

To further assess the 3D plausibility of the generated conformations, we evaluated the generated molecules using PoseBusters.^36^ Across the four targets, PharmaDiff achieved a higher PBR of (41.57 ± 18.35%), compared to (29.69 ± 21.99%) for DiffSBDD, indicating that a larger fraction of its generated molecules satisfy all structural, geometric, and chemical validity criteria simultaneously. We also note that the fraction of molecules satisfying all PoseBusters validation checks generally decreases as the number of pharmacophore features increases. This trend is expected, as molecules generated from more complex pharmacophore hypotheses must simultaneously satisfy a larger set of geometric and chemical constraints, making it more challenging to produce conformations that pass all validation criteria.

### Case Study: Demonstration of PhamaDiff in DDR1 Inhibitors Design

Discoidin domain receptor 1 (DDR1), a kinase target involved in fibrosis and multiple cancers, making it a crucial therapeutic target. Therefore, the discovery of structurally diverse DDR1 inhibitors is essential to expand chemical space, optimize drug-like properties, and facilitate broader therapeutic exploration. Thus, we applied PharmaDiff to generate molecules guided by a previously reported pharmacophore hypothesis^44^ adopted by the GENTRL model to generate active compounds using key interaction features within the ATP-binding site, including a hydrogen bond acceptor near the hinge region, an aromatic or hydrophobic linker in the central binding site, and a hydrophobic moiety close to the DFG motif. To reconstruct the atom-based representation of the pharmacophore hypothesis, the structural coordinates were derived from the DDR1–inhibitor cocrystal structure (PDB ID:5BVN).^45^ A total of 10,000 pharmacophore-conditioned molecules were generated by PharmaDiff using this DDR1 pharmacophore hypothesis, and invalid molecules and molecules containing fewer than 30 heavy atoms were removed to maintain structural complexity consistent with known DDR1 inhibitors.

After filtering, the remaining generated compounds were compared to DDR1 active molecules from ChEMBL (activity ≤1 μ M) using Tanimoto similarity^31,32^ calculated from Morgan fingerprints31 (radius = 2, 2048 bits). For each top-ranked generated molecule, the most similar known DDR1 inhibitor was identified. These known inhibitors, representing the highest similarity matches to the generated compounds, were docked into the DDR1 crystal structure (PDB ID:5BVN) using MAESTRO Glide.^46,47^ This set served as a baseline for the workflow and provided comparative scores for the generated molecule set. As expected, the known active molecules produced many reasonable poses with minor clashes that were resolved using MMGBSA^48^ with a protein flexibility radius of 6 Å. The top 8 poses where all clashes are resolved are shown (Figure S2). This reference set was highly enriched in alkyne examples with occasional alternative amide linkers. Closer examination of one of the optimized poses using Desmond MD^49^ revealed a stable pose with no high-energy torsions, indicating a favorable protein–ligand complex for this active comparator molecule (Figure S1). Additionally, WaterMap^50^ demonstrated both good coverage of the high-energy waters in the apo map and minimal high-energy waters within the holo map (Figure S4). Lastly, analysis of the conformational strain of the MMGBSA optimized ligand binding pose was performed using FreeForm from OpenEye.^51^ Moderate global strain was recorded for this molecule in the MMGBSA optimized pose (Figure S5). Taken together, known active molecules with similarity to the generated molecules gave reasonable docking poses (typical interaction fingerprints within the pocket) following docking and MMGBSA minimization. The poses were stable, given the short MD simulation times (20 ns), with good coverage of apo waters and minimal high-energy waters in the holo water map, with low to moderate global conformational strain energy for the MMGBSA optimized ligand docking pose. No further refinement of the pose was investigated.

Molecules generated using PharmaDiff showed high diversity from the known active reference molecules with a max Tanimoto similarity of 0.35. The most similar generated molecules were docked into 5BVN and minimized using MMGBSA. Given the structural diversity of these molecules relative to known DDR1 binders, some may represent promising starting points for further optimization and design before they can be reduced to practice in the lab. However, there was a sufficient number of molecules where the overall fit in the pocket was very reasonable, with many typical hinge binding elements (pyridine, pyrimidine rings) and some less conventional hinge binding elements (nitrile, oxygen atom of oxadiazole ring) directly out of docking. It is important to note that there are no known structures where a nitrile serves as a hinge binder in a kinase, meaning this pose could be suspect. Two representative molecules (molecule 586 and molecule 1602) were selected for further analysis using WaterMap, induced-fit docking molecular dynamics (IFD-MD), and molecular dynamics (MD) simulations.

Desmond MD analysis of the MMGBSA optimized poses for representative molecules 1602 (typical hinge interaction) and 586 (atypical hinge interaction) showed somewhat less stable profiles (Figure 4). To confirm these poses, both molecules were taken into IFD-MD analysis to see if they would result in a more stable pose. 586, as expected, minimized into a new pose with a more typical hinge interaction through the pyrazole aza nitrogen, while 1602 moved into a direct interaction with the hinge rather than a water-mediated interaction, as seen upon simple docking. Further analysis of the IFD-MD-generated poses demonstrated moderately stable contacts during MD simulations, typical water maps with good coverage of high-energy waters in the apo form, and minimal high-energy waters in the holo form with low to moderate global strain energies for the bound conformation, particularly for 1602 (Figure 5).

**Figure 4:**
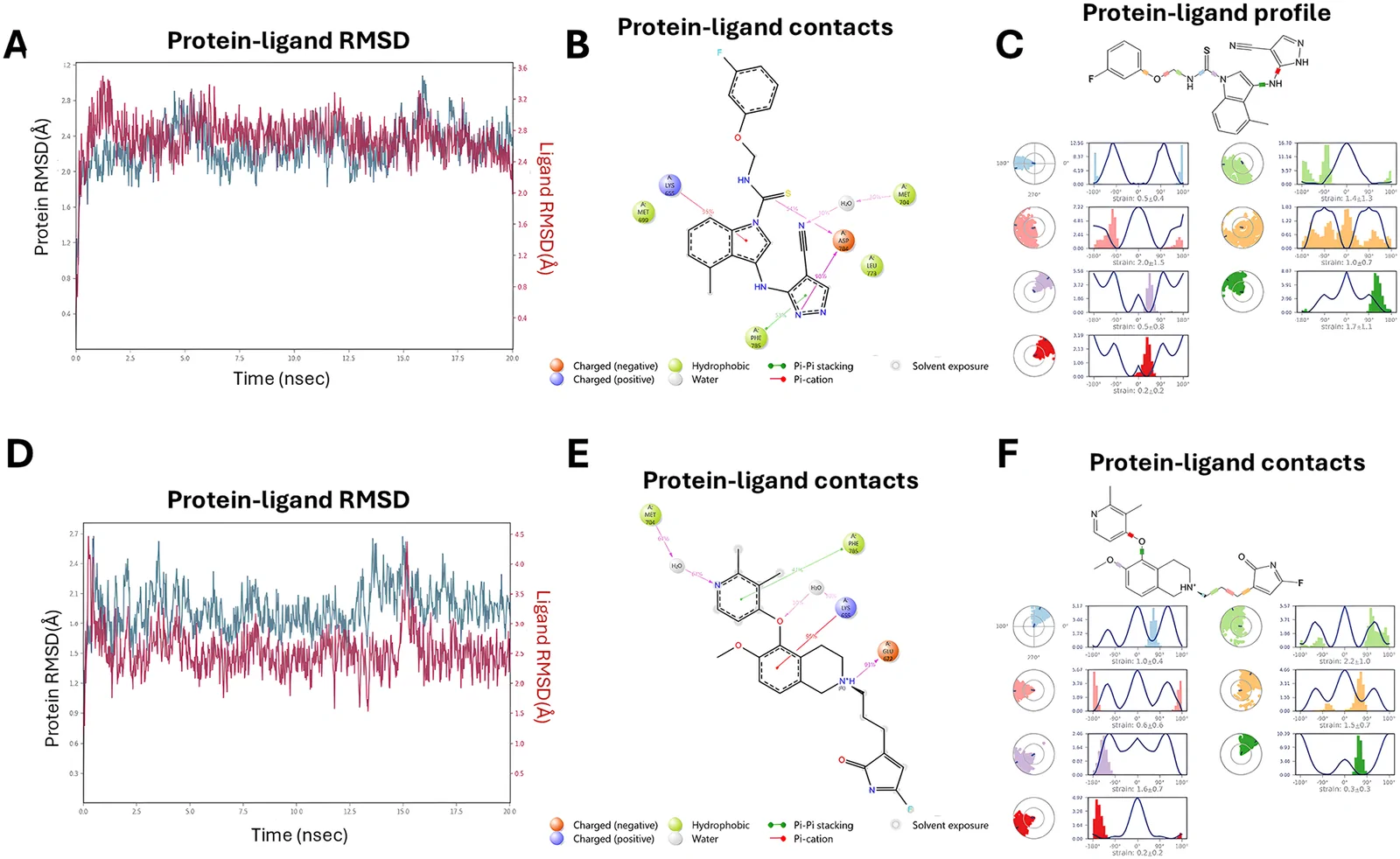
Molecular dynamics (MD) simulations (20 ns) of the docked and MMGBSA-optimized PharmaDiff-generated compounds: Compound 586 (A–C) and Compound 1602 (D–F). Panels A and D show the RMSD of the protein–ligand complex; panels B and E show ligand–protein interaction contacts; and panels C and F show the ligand torsional profiles.

**Figure 5:**
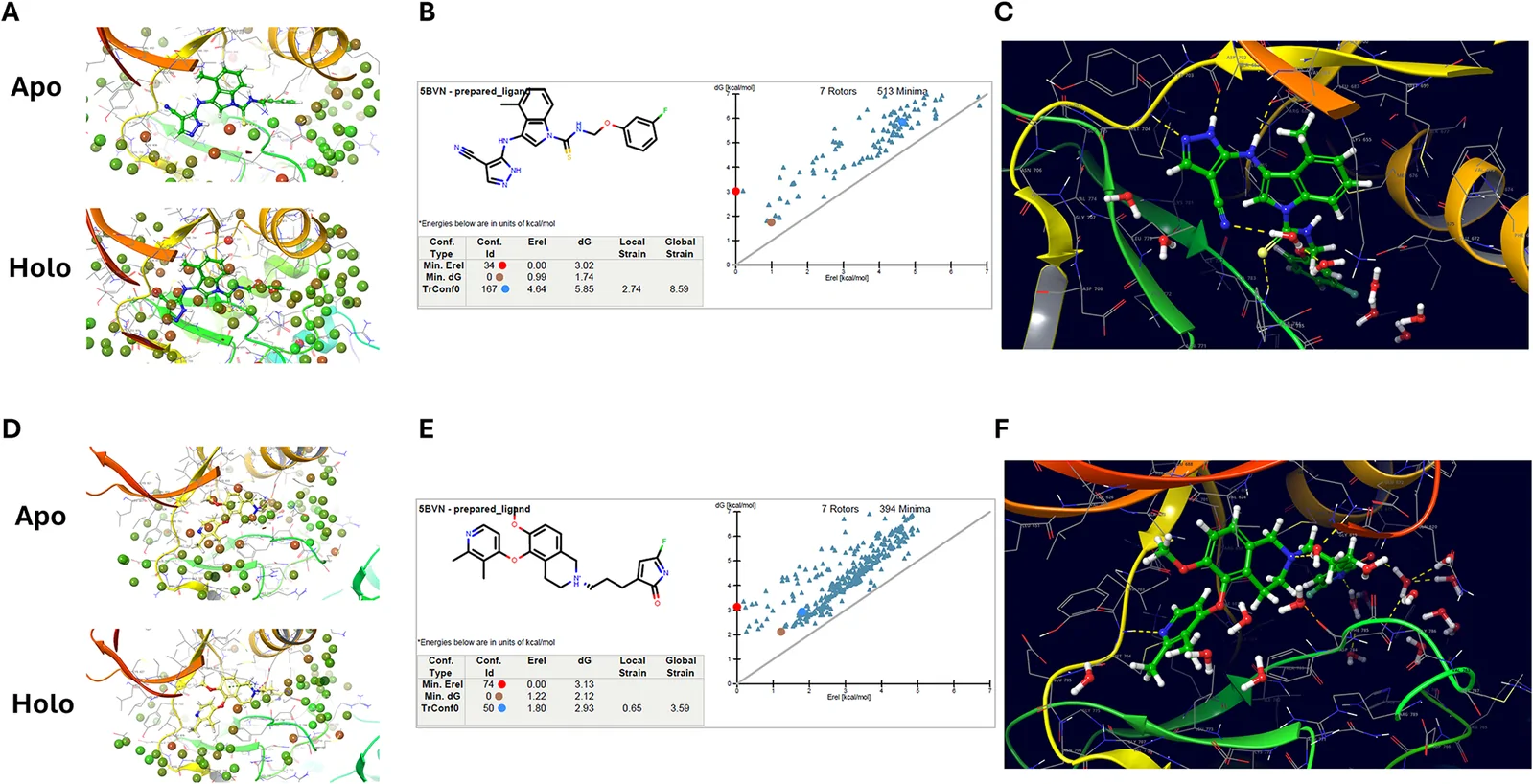
Binding site analysis and optimized ligand poses for two representative PharmaDiff-generated molecules. Panels A–C correspond to Molecule 586, and panels D–F correspond to Molecule 1602. (A, D) Apo and Holo structure of the DDR1 binding pocket with WaterMap hydration site analysis. (B, E) FreeForm global strain energy. (C, F) IFD-MD optimized ligand poses showing hinge-binding interactions in the ATP-binding pocket.

In summary, compounds generated for DDR1 kinase using pharmaDiff guided by a DDR1 pharmacophore^44^ displayed typical hinge binding elements, Type II frameworks with unoptimized back pocket features, and similar overall fit in the pocket with regard to comparisons to known DDR1 binders. The hinge interactions ran from typical (pyridine aza nitrogen of 1602) to less typical (donor–donor–acceptor motif of pyrazole moiety of 586), and any odd docking poses were easily assessed with IFD-MD. While these molecules are still more inspirational than perfectly designed, the necessary changes are minimal and, in some cases, could be resolved through single-atom corrections. For example, the elimination of nuisance groups such as hemiaminal and thiourea moieties in 586 would yield a more stable/drug-like molecule.

### Case Study: Design of BBB-Permeable PI3K_α_ Inhibitors via Pharmacophore-Guided Generation

Phosphoinositide 3-kinase α (PI3Kα) plays a central role in oncogenic signaling and is frequently activated in solid tumors such as breast, colorectal, and brain cancers. Its dysregulation promotes tumor proliferation, survival, and resistance to targeted therapies. Despite the development of several PI3Kα inhibitors, their effectiveness against brain cancers remains limited due to poor penetration of the blood–brain barrier (BBB). To overcome this limitation, we implemented a scaffold hopping strategy aimed at designing PI3Kα inhibitors with enhanced central nervous system (CNS) drug-like properties.^52^ A structure-based pharmacophore model was derived from the cocrystal structure of *Taselisib* bound to PI3Kα (PDB ID:8EXL8EXL) using PLIP.^53^ Guided by this pharmacophore hypothesis, we generated a library of 5000 molecules through scaffold replacement, retaining the essential interaction-driving pharmacophore features while introducing scaffold diversity. In particular, we aimed to replace *Taselisib*’s hydrophilic heterocycles, specifically the pyrazole and the triazole rings, which contribute significantly to its topological polar surface area (TPSA) and may hinder BBB permeability, with less polar scaffolds that preserve the spatial arrangement of key pharmacophoric groups. As illustrated in Figure 6, PharmaDiff successfully generated molecules with chemically diverse scaffolds that preserved the 3D spatial alignment of the pharmacophore hypothesis with the reference ligand, *Taselisib*.

**Figure 6:**
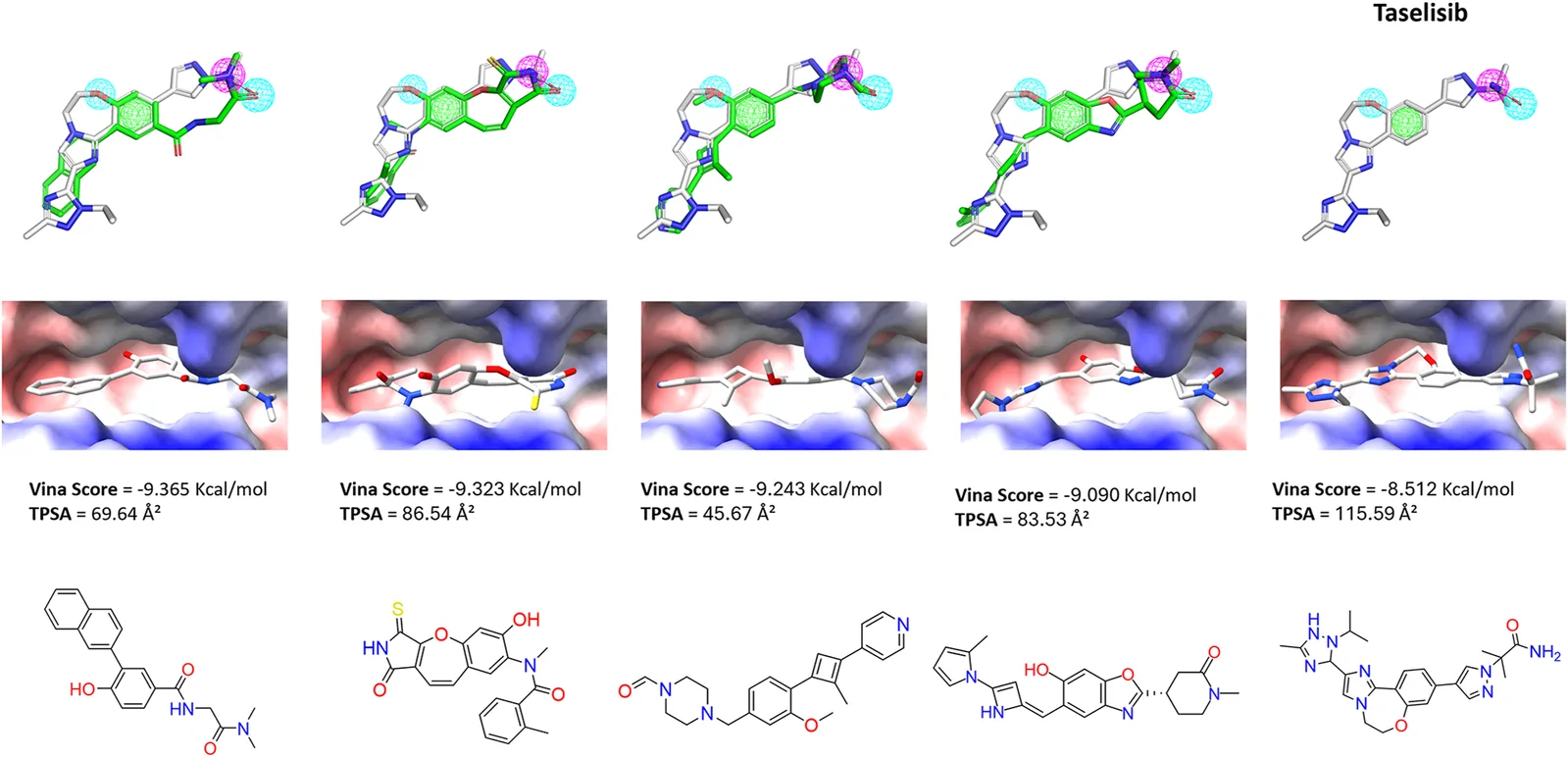
Top 4 molecules ranked by docking score, showing chemically diverse scaffolds generated by PharmaDiff. The top row illustrates the spatial alignment of pharmacophoric features in the generated ligands (green) to the pharmacophore derived from *Taselisib* (gray). Pharmacophore features are color-coded as follows: hydrogen bond acceptors in cyan, hydrophobes in green, and hydrogen bond donors in magenta. The middle row shows the docked poses of the generated ligands within the PI3Kα binding pocket. The bottom row displays the corresponding 2D chemical structures, highlighting scaffold diversity among the top-scoring candidates.

Unlike the DDR1 case study, which was designed to demonstrate in-depth validation of selected PharmaDiff-generated compounds using several downstream computational methods, the PI3Kα case study was intended to demonstrate pharmacophore-guided scaffold hopping for the design of BBB-permeable inhibitors. Therefore, we employed a more computationally efficient workflow based on large-scale molecule generation, CNS-oriented property filtering, and docking-based prioritization, rather than the extensive postdocking analyses performed in the DDR1 study.

To prioritize BBB-permeant candidates, we applied CNS drug-like property filters based on molecular weight (≤400 Da), log *P*(2–4), topological polar surface area (TPSA ≤ 90 Å^2^), hydrogen bond donors (≤2), and acceptors (≤6). Molecular descriptors were computed using RDKit. This filtering step yielded 441 compounds, 433 of which were unique. All filtered compounds were docked to the PI3Kα structure (8EXL) using AutoDock Vina, focusing on the *Taselisib* binding site. The best-scoring pose for each molecule was retained for further analysis. Of these, 113 compounds (26.10%) achieved docking scores below −8.0 kcal/mol, and **39 compounds (9.01%)** surpassed the reference ligand *Taselisib*, which scored −8.512 kcal/mol. These results demonstrate the potential of pharmacophore-guided scaffold hopping to identify novel PI3Kα inhibitors with improved BBB permeability and potent binding affinity. As illustrated in Figure 6, PharmaDiff was able to generate diverse scaffolds, allowing us to explore alternative core structures while preserving key pharmacophoric interactions essential for PI3Kα binding. However, it is important to note that while several generated molecules exhibit promising binding characteristics, additional optimization or rigorous filtration may be required to ensure synthetic accessibility and chemical stability prior to experimental validation.

## Discussion

PharmaDiff demonstrates strong performance in pharmacophore-conditioned generation, achieving higher pharmacophore matching accuracy than SMILES-based baselines and outperforming prior 3D structure-based generative models in docking scores. However, the model still faces challenges, particularly in the inpainting process, where atoms are modified to align with predefined pharmacophoric features. While this approach can retain important properties such as aromaticity or the presence of hydrogen bond acceptors, it may occasionally lose key pharmacophoric features when the local atomic context changes. Subtle structural modifications can disrupt hydrogen bonding, hydrophobic interactions, or other noncovalent forces essential for target binding and biological activity. Although PharmaDiff implicitly incorporates pharmacophoric feature types as contextual input during generation, these features are not explicitly reinforced. Despite the strong performance achieved through this implicit integration, evidenced by an average **PMR** of 0.6990, future work could explore explicitly enforcing the retention of pharmacophoric features in the loss function through a differentiable, feature-aware model to better preserve those critical for target interactions.

An additional challenge common to atom-level inpainting approaches, including PharmaDiff and similar models,^15,54^ is the generation of disconnected molecular structures, particularly when handling complex pharmacophores with several features and thus a large number of associated inpainted atoms. The common practice of retaining only the largest connected fragment to maintain connectivity can lead to the exclusion of pharmacophore-associated atoms, potentially resulting in the loss of critical functional groups. As a result, the generated molecules may lack features essential for bioactivity. Future improvements could focus on enhancing connectivity within the inpainting process through stricter structural constraints, energy-guided sampling, or postprocessing optimization and energy-minimization techniques that preserve both the overall molecular integrity and key pharmacophoric elements.

Another limitation of PharmaDiff is that it requires predefined 3D pharmacophoric atoms as conditioning input; however, these constraints correspond only to the pharmacophoric atoms and their spatial arrangement rather than a complete molecular scaffold. Many pharmacophore features, such as hydrogen bond donors, hydrogen bond acceptors, and hydrophobic features, are represented by only a small number of atoms and can be satisfied by a wide range of chemically distinct functional groups. As a result, PharmaDiff retains flexibility in determining the surrounding chemical environment and overall molecular scaffold while satisfying the desired pharmacophoric constraints. Furthermore, this limitation can be partially mitigated by presampling diverse atoms or molecular fragments that satisfy the same pharmacophore hypothesis, as demonstrated in the CDK2 fragment-sampling case study in the Supporting Information, where diverse molecular substituents were used as conditioning inputs under the same pharmacophore hypothesis.

A further limitation of PharmaDiff is its dependence on the availability of a pharmacophore hypothesis to guide molecular generation. The pharmacophore models used here were derived from crystal structures bound to ligands of DDR1 and PI3Kα, and while pharmacophore hypotheses can also be built from known active compounds or predicted protein–ligand complexes using tools such as AlphaFold 3,^55^ Boltz-2,^56^ and Protenix,^57^ PharmaDiff is less applicable to novel targets lacking reliable 3D structural data, targets for which only apo structures are available, or systems for which predicted protein–ligand complexes are unreliable. Therefore, applying PharmaDiff in these settings may require additional experimental data to construct a robust pharmacophore hypothesis.

Despite this limitation, PharmaDiff can be incorporated into broader pharmacophore-guided drug discovery pipelines. For example, pharmacophore modeling approaches such as PharmacoNet^58^ may be used to derive pharmacophore hypotheses from known ligands or protein structures before molecular generation. In addition, pharmacophore mapping frameworks such as DiffPhore^59^ could be used to assess how well generated molecules match the conditioned pharmacophoric features. Pharmacophore-constrained docking and virtual screening methods may also be applied as downstream filters to prioritize promising candidates. Future studies may explore combining PharmaDiff with these complementary approaches to create more integrated pharmacophore-guided drug discovery pipelines.

Another limitation of the current PharmaDiff framework is that it is primarily designed for noncovalent binders and does not explicitly model covalent bond formation or metal coordination. Extending the model to covalent inhibitors would require incorporating information about reactive warheads, potentially as an additional pharmacophore feature type. Similarly, the design of metalloenzyme inhibitors would require explicit consideration of the coordinating groups, with their preferred coordination geometry. Resources such as the Metalloenzyme Data Bank and Analysis (MeDBA)^60^ could provide useful information for extending PharmaDiff to better represent these specialized interactions.

From a practical drug discovery perspective, PharmaDiff is intended to support the design stage of a broader Design–Make–Test (DMT) cycle. After molecule generation, several filtering and prioritization steps can be applied before experimental validation. For example, Pan-Assay Interference Compounds (PAINS)^61^ and Rapid Elimination of Swill (REOS)^62^ filters can be used to remove compounds containing undesirable or potentially problematic substructures that may interfere with biological assays. Retrosynthetic planning tools, such as AiZynthFinder^63^ and ASKCOS,^64^ can then be employed to assess synthetic accessibility and identify compounds that are more feasible to synthesize. In addition, analog searching can help identify commercially available compounds with similar chemical scaffolds, enabling rapid experimental evaluation without requiring immediate synthesis. Methods such as SynFormer^65^ provide another complementary approach by generating synthetically accessible analogs of a query molecule, which may facilitate lead optimization while preserving key structural and pharmacophoric features.

Together, these complementary approaches can help filter generated candidates to a smaller set of synthesizable molecules while maintaining the desired pharmacophoric characteristics. Further integration of pharmacophore-guided generation with synthesis-aware molecular generation represents an interesting direction for future work.

## Conclusions

We presented PharmaDiff, a 3D diffusion-based model for pharmacophore-conditioned molecular generation. PharmaDiff outperformed baselines, including PGMG, TransPharmer, REINVENT4, ShEPhERD and Phoregen in pharmacophore matching accuracy, demonstrating the benefits of direct 3D conditioning and Euclidean space matching. In structure-based design tasks, it also achieved superior docking scores and pharmacophore alignment compared to DiffSBDD, while maintaining favorable synthetic accessibility and molecular diversity. These results highlight PharmaDiff’s potential as a powerful tool for de novo drug design.

## Data and Software Availability

The source code of this study is freely available at GitHub (https://github.com/AmiraAlakhdar/PharmaDiff). The raw and processed data for the GEOM-Drugs dataset used in this study are publicly available on Zenodo at: https://zenodo.org/records/15740452. This includes both the original molecular structures and the pharmacophore-annotated formats used in our experiments.

## Supplementary Material



## Notes

Ablation study of inpainting, cross-attention, and pharmacophore-specific loss terms; pharmacophore fragment sampling for CDK2 inhibitors and pharmacophore-guided ligand generation; Glide docking protocol for DDR1 inhibitors; docking poses of known DDR1 inhibitors; Desmond molecular dynamics analysis; WaterMap analysis; and FreeForm strain-energy analysis (PDF)
