# Supplementary material for: Pharmacophore-Conditioned Diffusion Model for Ligand-Based De Novo Drug Design
Source: J Chem Inf Model. 2026 Oct 26;66(21):13768–82. doi: 10.1021/acs.jcim.6c01046 (PMC13753508; doi:10.1021/acs.jcim.6c01046)
Supplement: Supplementary file 1 [file ci6c01046_si_001.pdf]

Supporting Information: Pharmacophore-Conditioned Diffusion Model for Ligand-Based *De Novo* Drug Design

Amira Alakhdar,† Barnabas Poczos,‡ Newell Washburn,*,†,¶ and Kevin Cusack§

†*Department of Chemistry, Carnegie Mellon University, Pittsburgh, PA, 15213, USA*

‡*Department of Machine Learning, Carnegie Mellon University, Pittsburgh, PA, 15213, USA*

¶*Department of Biomedical Engineering, Carnegie Mellon University, Pittsburgh, PA, 15213, USA*

§*AbbVie Bioresearch Center, Worcester, MA, 01605, USA*

E-mail: washburn@andrew.cmu.edu

Ablation study: impact of inpainting and cross attention on pharmacophore-conditioned generation

To evaluate the contribution of the key conditioning mechanisms in PharmaDiff, we performed an ablation study comparing the full model against three variants: (i) a model without inpainting, (ii) a model without the cross-attention mechanism, and (iii) a model without the pharmacophore-specific loss adjustment. In the full model, inpainting provides explicit spatial and chemical conditioning by fixing the positions and identities of pharmacophore-associated atoms throughout the denoising process. In the no-inpainting variant, only the pharmacophore feature types (e.g., hydrogen bond donor, hydrogen bond acceptor, hydrophobe, and aromatic features) were provided, without positional or fragment-level information. The no-cross-attention variant retained inpainting but removed the interaction between molecular and pharmacophore embeddings. Finally, the no-loss-adjustment variant

removed the pharmacophore-specific loss terms while retaining all other components of the model.

As shown in Table S0, removing inpainting resulted in the largest performance degradation across all metrics. Validity decreased from 0.8789 to 0.6494, while pharmacophore matching performance dropped substantially, with MS decreasing from 0.8995 to 0.6899 and PMR decreasing from 0.6823 to 0.2105. These results demonstrate that explicit spatial conditioning through inpainting is the primary mechanism enabling the model to satisfy the desired pharmacophore hypothesis.

Removing the cross-attention module produced a more moderate reduction in performance. While validity remained largely unchanged (0.8682), MS and PMR decreased to 0.8335 and 0.6423, respectively. This suggests that cross-attention provides complementary guidance by allowing generated molecular representations to dynamically incorporate pharmacophoric information, although its contribution is smaller than that of inpainting.

The removal of the pharmacophore-specific loss resulted in only modest reductions in pharmacophore matching performance (MS: 0.8995 to 0.8759; PMR: 0.6823 to 0.6557). This observation is expected because pharmacophore-associated atoms are already included in the overall molecular generation loss. Nevertheless, the additional pharmacophore-specific loss consistently improved adherence to the conditioned pharmacophore hypothesis by increasing the supervision signal associated with pharmacophore-constrained atoms.

Overall, these results indicate that inpainting is the dominant contributor to pharmacophore-guided generation, while cross-attention and the pharmacophore-specific loss provide complementary improvements that further enhance pharmacophore matching performance.

Table S0: Ablation study comparing PharmaDiff with and without inpainting. Results are reported over 1,024 generated molecules.

**Ablation Study: Effect of Inpainting and cross attention**					
Model	Validity ↑	Uniqueness ↑	Novelty ↑	MS ↑	PMR ↑
PharmaDiff (3D)	**0.8789**	0.9900	1.00	**0.8995**	**0.6823**
PharmaDiff (3D, No Inpainting)	0.6494	**1.00**	1.00	0.6899	0.2105
PharmaDiff (No Cross Attention)	0.8682	0.9933	1.00	0.8335	0.6423
PharmaDiff (No Loss Adjustment)	0.9189	0.9957	1.0000	0.8759	0.6557

Case Study: Pharmacophore-Guided Fragment Sampling in CDK2 Inhibitors

Cyclin-dependent kinase 2 (CDK2) regulates the G1/S phase transition of the cell cycle and is frequently dysregulated in a variety of cancers, making it a well-studied therapeutic target. *Roscovitine*, a purine-based ATP-competitive inhibitor, binds to CDK2 as captured in the co-crystal structure (PDB ID 2A4L).[1] By inspecting this structure with PLIP,[2] we extracted a set of pharmacophore features that reflect key ligand-protein interactions.

To evaluate whether pharmacophore conditioning restricts scaffold diversity, we performed a pharmacophore-guided fragment sampling experiment. First, 3D pharmacophore points were extracted from the ligand–protein complex. We then sampled diverse atoms and molecular fragments corresponding to each pharmacophore type, including hydrogen bond donor and acceptor groups, aromatic heterocycles such as pyridine, pyrimidine, imidazole, and furan, as well as hydrophobic fragments including benzene rings, cycloalkanes (e.g., cyclobutane and cyclopropane), and branched alkyl groups such as isopropyl and tert-butyl. The 3D geometries of all fragments were minimized using the MMFF94 force field. Finally, PharmaDiff was conditioned on the extracted pharmacophore together with the sampled 3D fragment inputs to generate 200 candidate molecules. Representative pharmacophore-aligned generated ligands are shown in Figure S1.

Glide Docking Protocol of DDR1 inhibitors

A Glide Grid docking file was created from the 5BVN crystal structure, using the Grid Generation GUI within MAESTRO. The Grid file included constraints for Met704, Thr701, Glu672, Asp784 and a structural water. All constraints were checked in the Receptor tab with a setting of "Match at least one" constraint. Docking and subsequent evaluation of conformation, water energetics and stability of pose was benchmarked by evaluating a set of known DDR1 inhibitory compounds from the ChEMBL dataset (as shown in Supplementary Figures S2–S5).

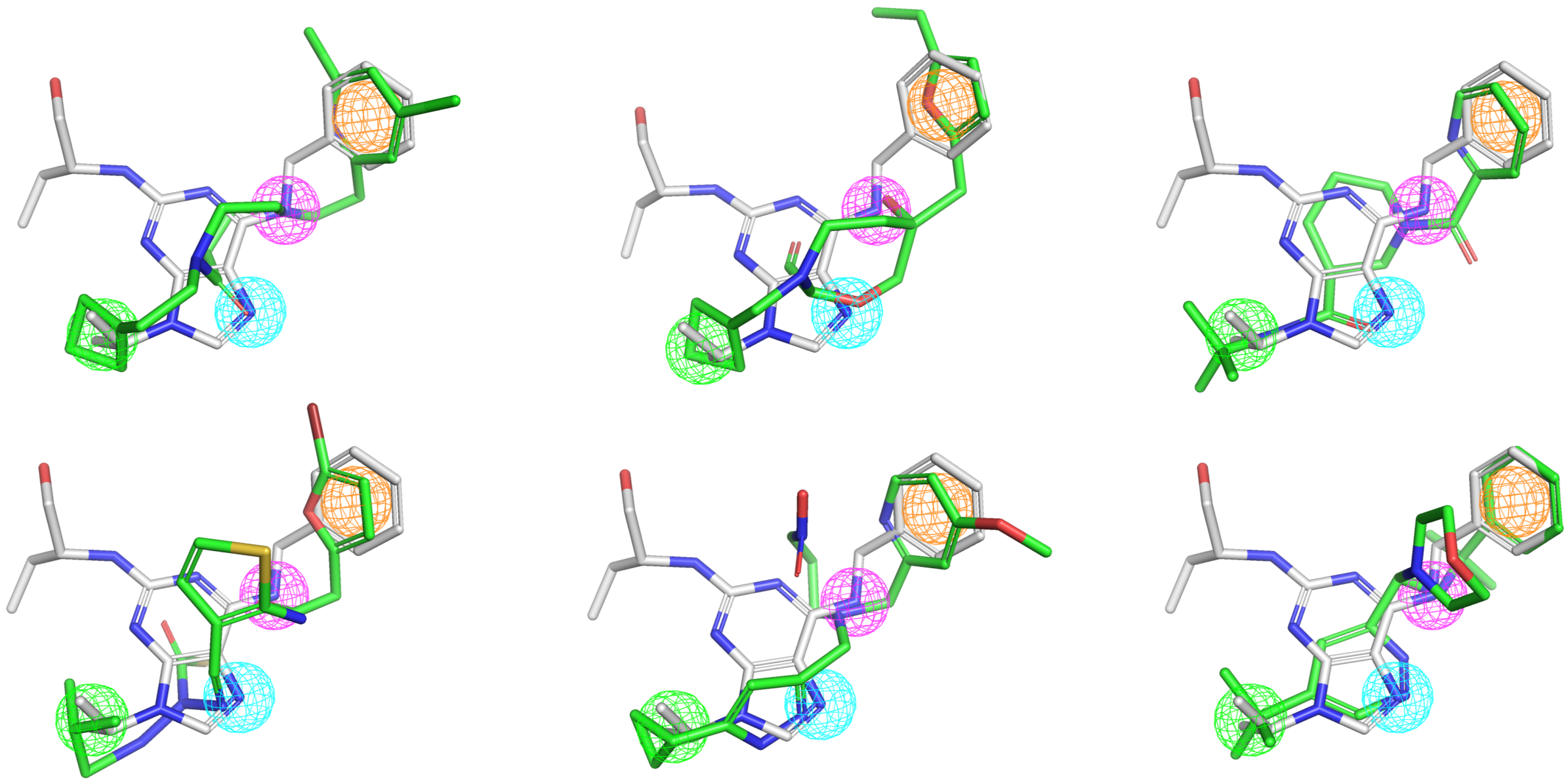

Figure S1: pharmacophore-aligned generated ligands (green) compared to the reference CDK2 inhibitor ligand *Roscovitine* (grey). Pharmacophore features are color-coded as follows: hydrogen bond acceptors in cyan, hydrophobes in green, and aromatic groups in orange and hydrogen bond donors in magenta.

References

(1) Azevedo, W. F. D.; Leclerc, S.; Meijer, L.; Havlicek, L.; Strnad, M.; Kim, S.-H. Inhibition of cyclin-dependent kinases by purine analogues: crystal structure of human CDK2 complexed with roscovitine. *European Journal of Biochemistry* **1997**, *243*, 518–526.

(2) Adasme, M. F.; Linnemann, K. L.; Bolz, S. N.; Kaiser, F.; Salentin, S.; Haupt, V. J.; Schroeder, M. PLIP 2021: expanding the scope of the protein-ligand interaction profiler to DNA and RNA. *Nucleic Acids Research* **2021**, *49*, W530–W534.

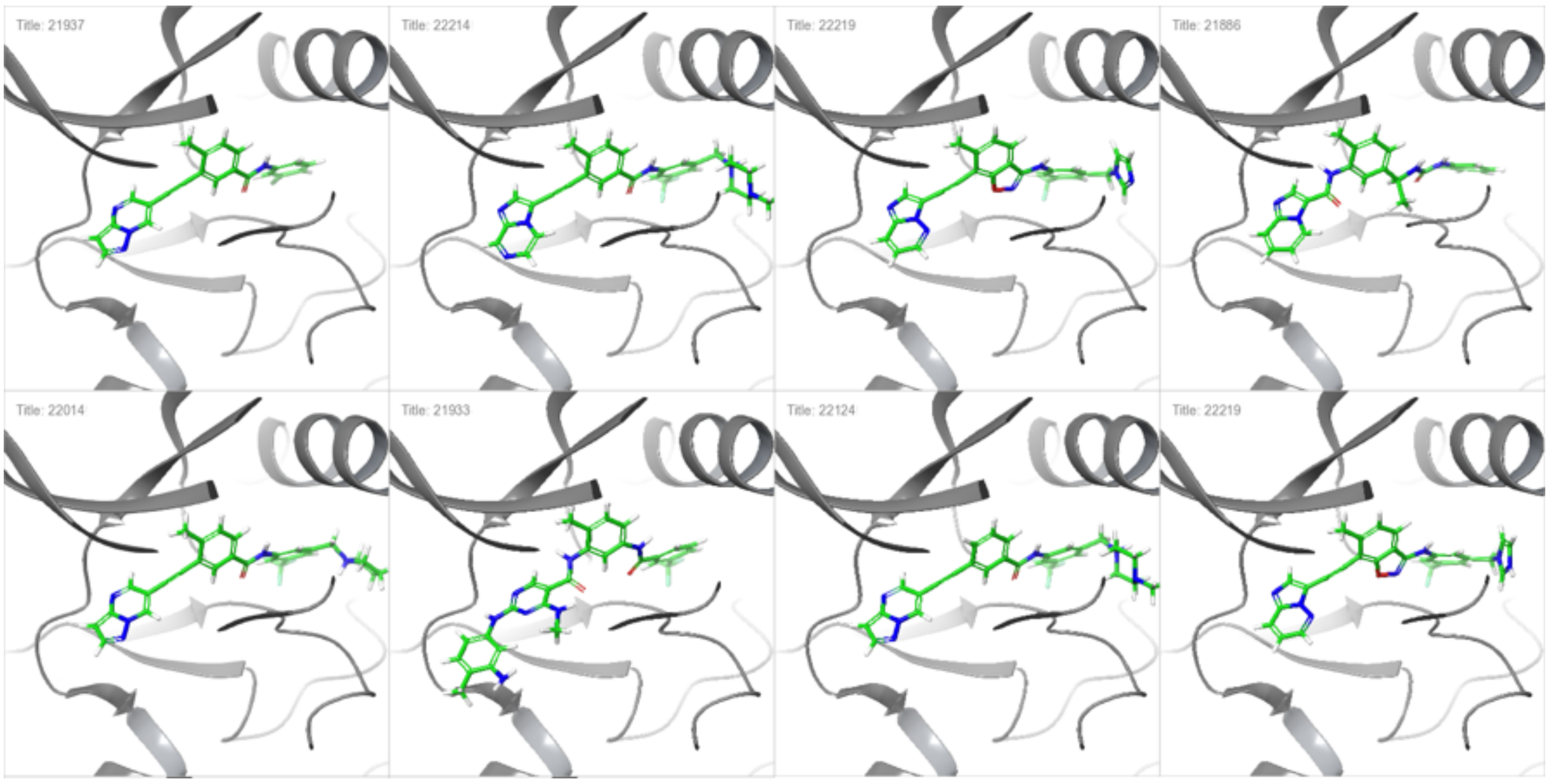

Figure S2: Docking poses for most similar known DDR1 inhibitors

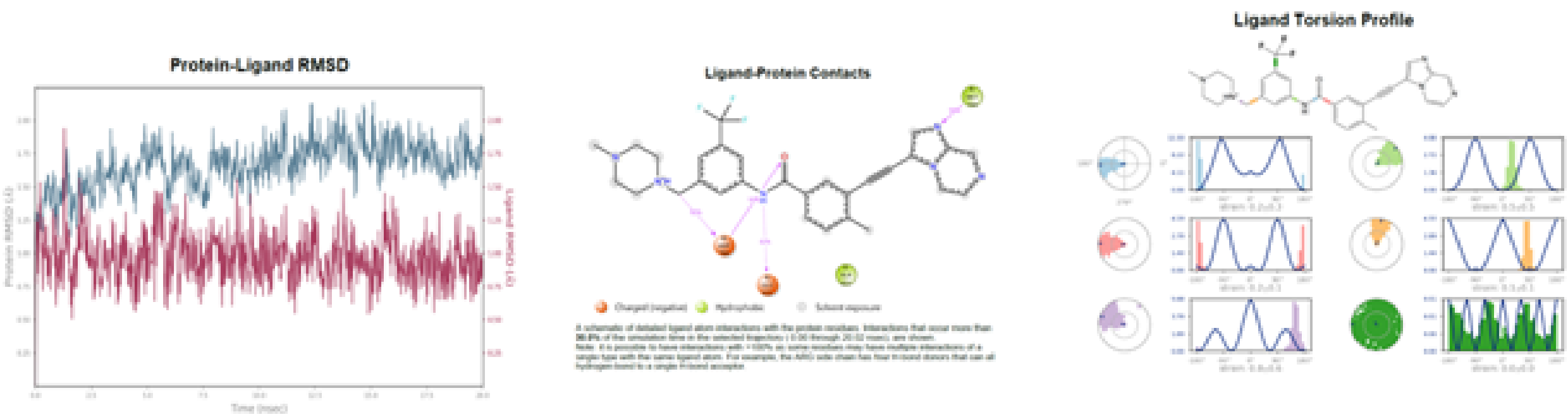

Figure S3: Desmond MD summary from 20ns simulation. Left: Stability of protein and ligand throughout simulation; Center: Ligand Interaction Diagram; Right: Ligand Torsion Profile

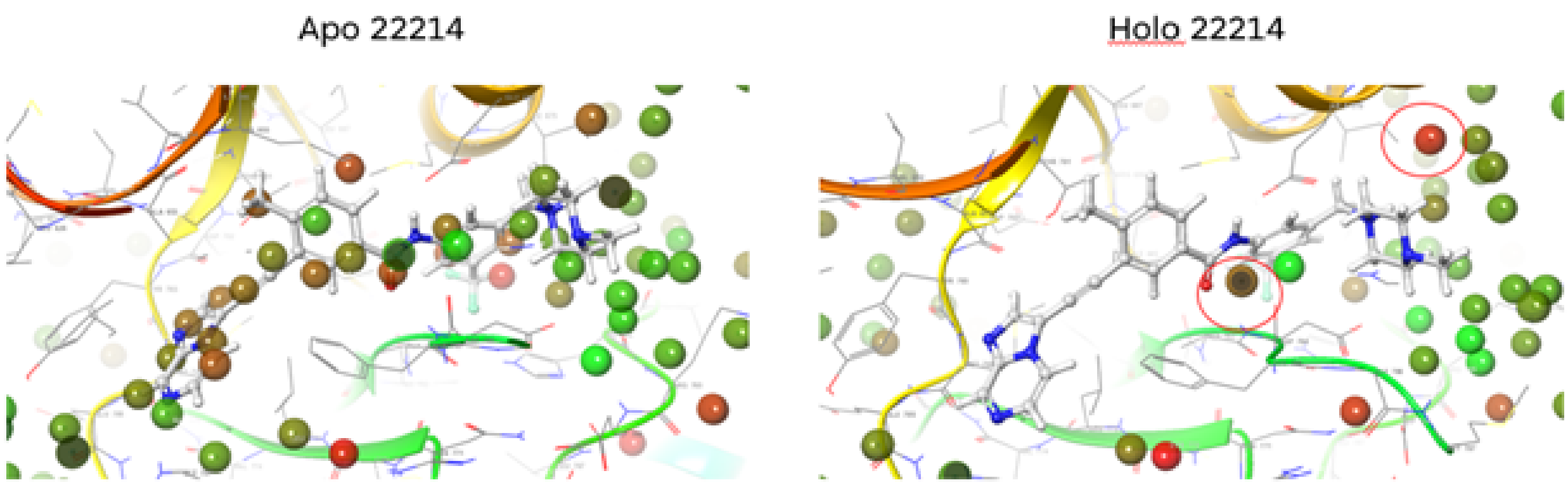

Figure S4: Apo and Holo water maps for Molecule 22214 bound to DDR1 after MMGBSA minimization (5BVN Grid)

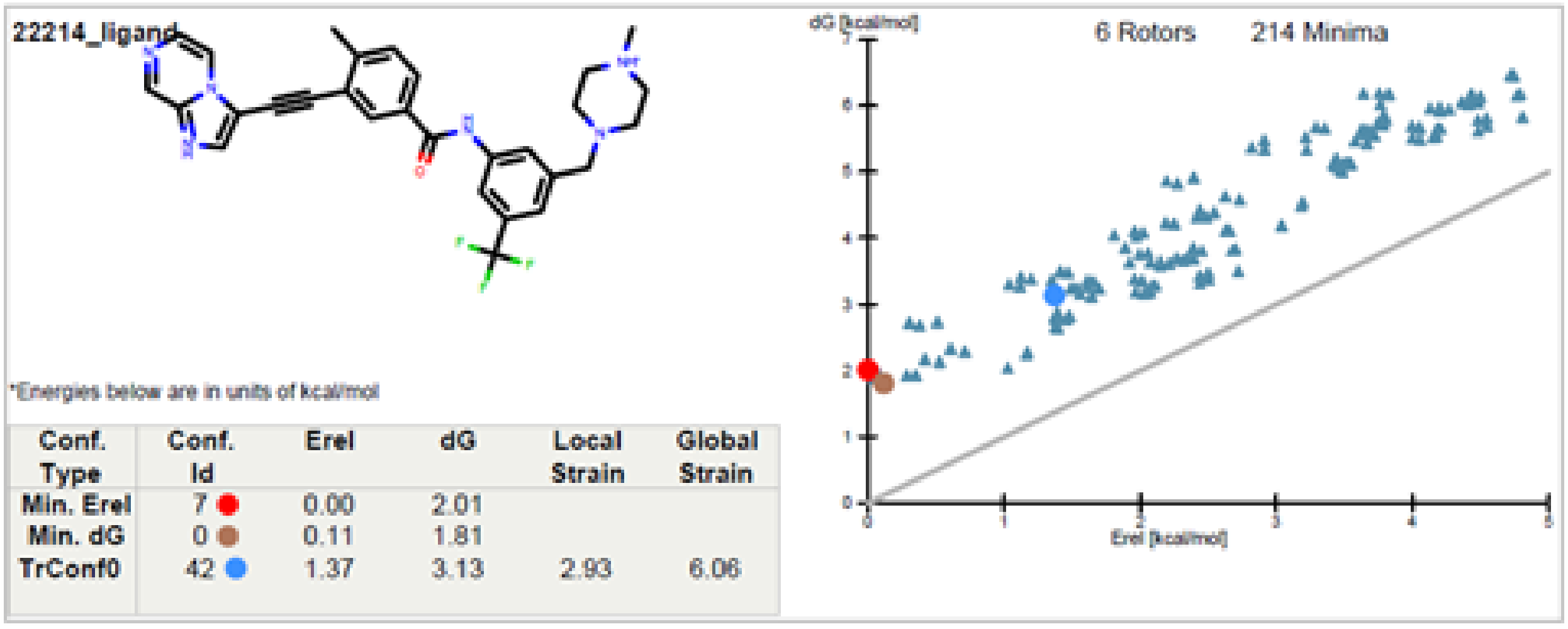

Conf. Type	Conf. Id	Erel	dG	Local Strain	Global Strain
Min. Erel	7	0.00	2.01		
Min. dG	0	0.11	1.81		
TrConf0	42	1.37	3.13	2.93	6.06

Figure S5: FreeForm global strain energy Molecule 22214
